# Higher-dimensional ordination analysis teases out impacts of *Bradyrhizobium* bioaugmentation on native soil microbial communities

**DOI:** 10.1128/spectrum.02880-25

**Published:** 2026-02-18

**Authors:** Hiromi Kato, Manabu Itakura, Shusei Sato, Rota Wagai, Kiwamu Minamisawa

**Affiliations:** 1 Graduate School of Life Science, Tohoku University 13101, Sendai, Japan; 2 National Agriculture and Food Research Organization (NARO), Institute for Agro-Environmental Sciences (NIAES) 13589, Tsukuba, Ibaraki, Japan; Instituto de Ecología, A.C. (INECOL), Pátzcuaro, Michoacán, Mexico

**Keywords:** microbial inoculants, soil microbiome, dimensionality reduction, *nosZ*

## Abstract

Microbial inoculants are increasingly being used to enhance plant growth and soil environments, yet their ecological effects on native microbial communities remain unclear. We investigated the long-term dynamics of soybean-nodulating *Bradyrhizobium* strains carrying the *nosZ* gene encoding N_₂_O reductase in microcosms comprising three different soils. Analysis of 16S rRNA amplicons revealed that bacterial community compositions were primarily driven by soil type and time, with inoculation having only minor effects. In contrast, *nosZ* clade I amplicon analysis showed high survivability of the dominant *nosZ*-sequence groups corresponding to the inoculant strains, which accounted for 20%–50% of the total *nosZ* community even 249 days after inoculation; despite this dominance, the overall community structures of indigenous *nosZ*-harboring *Bradyrhizobium* remained largely unchanged. This observation was further supported by a permutational multivariate analysis of variance, which showed a 90% reduction in R^2^ when the inoculant sequence groups were excluded. Thus, it is possible to enhance N₂O-reducing function through inoculation without drastically disrupting the indigenous microbial community. To explore the landscapes of inoculation effects obscured in two-dimensional mapping, we applied dimensionality-reduction tools such as principal coordinates analysis beyond their typical use for visualization by extending analyses into higher-dimensional spaces. Varying the number of dimensions revealed that the signal-to-noise ratio and clustering tendency peaked at 4–10 dimensions, with further increases in dimensions leading to homogenization of community patterns. This intermediate dimensional space also revealed differences in community succession by soil type. These findings demonstrate that careful selection of dimensionality can enhance the discovery of ecological patterns.

**IMPORTANCE:**

Dimensionality reduction is widely used to visualize microbiome data in two- or three-dimensional ordination spaces. However, its application in higher-dimensional analysis remains underexplored. We highlighted the use of dimensionality reduction not only as a visualization tool, but as a means of projecting microbiome data into ordination spaces for geometric analyses, which are not limited to two or three dimensions. Communities were projected beyond three dimensions to examine how dimensionality affects evaluation of inoculation effects through geometric analyses. Our findings show that dimension selection strongly influences the ability to detect and resolve ecological signals, which were distorted in low-dimensional spaces or homogenized in extremely high-dimensional spaces. Intermediate dimensionalities better retained the spatial fidelity needed to resolve soil-dependent responses to inoculation. By enhancing the resolution of small but meaningful effects, this approach provides a robust framework for guiding strain selection, application strategies, and risk assessment in microbial inoculation studies.

## INTRODUCTION

The genus *Bradyrhizobium* includes numerous root-nodulating bacteria, some of which are known to establish symbiotic relationships with soybean plants, supplying ammonia through biological nitrogen fixation (1, 2). Such symbioses reduce the need for chemical nitrogen fertilizers and have positioned *Bradyrhizobium* as a model organism in agricultural microbial inoculation studies, prompting extensive research into its nitrogen fixation systems and symbiotic mechanisms (3). Numerous strains have been isolated worldwide, revealing considerable diversity in metabolic capacities and phylogenetic relationships (4).

Beyond symbiosis, the role of *Bradyrhizobium* in soil nitrogen cycling has gained attention. Organic nitrogen from nodules can be transformed via nitrification and denitrification, releasing nitrous oxide (N₂O), a potent greenhouse gas (5, 6). Some *Bradyrhizobium* strains harbor *nosZ* genes encoding N₂O reductase, which can facilitate decreased N₂O emissions (6, 7). For example, *B. ottawaense* SG09 shows greater N₂O reduction capacity than the well-studied *B. diazoefficiens* USDA110 (8, 9).

Although genetic studies have clarified the evolutionary basis of nitrogen fixation and symbiosis (4), the ecological behavior of *Bradyrhizobium* in bulk soil remains less well understood. Increasing evidence suggests that *Bradyrhizobium*, including non-symbiotic lineages, constitutes a major component of general soil microbial communities that potentially affects inoculant persistence and nodulation dynamics in the field (10, 11). Strain competitiveness likely depends not only on symbiotic capacity but also on traits such as metabolic versatility, environmental tolerance, and denitrification potential.

However, unintended ecological impacts of microbial inoculants on native soil communities are of concern (12). Although long-term field studies on *Bradyrhizobium* have shown that inoculated strains can survive for years (13), most studies have focused on strain recovery and nodulation, leaving community effects underexplored. In a field study in Scotland, where native *Bradyrhizobium* is scarce, the effects of *Bradyrhizobium* inoculation on soil bacterial communities were examined using 16S rRNA amplicon analysis and q-PCR (14). In a case from Japan, where native *Bradyrhizobium* is abundant, 16S-based surveys were supplemented with *nosZ* amplicon analyses to track inoculants (15); in that study, the effects of inoculation on 16S-rRNA diversity were found to be minor compared with land-use variation, whereas *nosZ*-based profiles sporadically detected inoculant strains. Together, these studies highlight both the value and limitations of field-based evaluations under complex ecological conditions. In open field studies, factors such as rainfall (16), soil heterogeneity (17, 18), host plant effects (14), and microbial immigration and emigration (19) complicate direct assessments of inoculant survivability and competitiveness with indigenous communities. To address these challenges, in our study, we employed two approaches: (i) using closed soil microcosm systems in the absence of plants to control migration and environmental conditions and (ii) narrowing the analytical scale from whole-community to functionally or taxonomically defined subcommunities.

To investigate the temporal dynamics of microbial community responses to perturbations such as microbial inoculation, researchers commonly first convert community composition data into dissimilarity matrices (e.g., Bray–Curtis, UniFrac) and then apply dimensionality-reduction methods such as principal coordinates analysis (PCoA) or non-metric multidimensional scaling (NMDS) to project the samples into two- or three-dimensional spaces for visualization (20, 21). These coordinate spaces enable geometric analyses (22, 23) that cannot be performed directly on the dissimilarity matrices. However, while dimensionality reduction is widely regarded as a visualization tool, its potential as a preprocessing step in higher-dimensional spaces remains underexplored (24). In this study, we applied dimensionality reduction to project microbiome data into ordination spaces with more than three dimensions. In these extended spaces, we used geometric analyses to evaluate community changes while assessing the fidelity of spatial structure across dimensionalities, with the goal of disentangling modest inoculation effects from background variation arising from soil differences and natural temporal change.

## MATERIALS AND METHODS

### Soils

The soils used in this study were obtained from the plow layer of three agricultural fields comprising two upland soils (ANT and AT) classified as Andosols and one paddy field soil (G) classified as a Gleysol. ANT and AT were collected from plots under long-term soybean/winter-wheat rotation, maintained at the experimental agricultural field of the National Institute for Agro-Environmental Sciences in Tsukuba, Ibaraki, Japan (36°01′26.4″N 140°06′41.6″E) (25–27). ANT was managed under no-till conditions with annual application of composted leaves, whereas AT was managed under conventional tillage conditions with annual application of inorganic nitrogen, phosphorus, and potassium. The G sample was collected from the Yawara Experimental Paddy Field of the National Agriculture and Food Research Organization (36°00′20″ N, 140°01′23″ E) (28), where no soybean cultivation has been recorded. AT and G samples were collected from a depth of 0–20 cm, while ANT samples were collected from a depth of 5–20 cm to exclude the surface organic-rich layer formed under long-term no-till management. The physicochemical properties of these soils were summarized in Table S1.

### Bacterial strains and cultivation

We used two high-N₂O-reducing strains, *Bradyrhizobium ottawaense* SG09 and a *nasS* mutant of *B. diazoefficiens* USDA110 (29), each chromosomally tagged with streptomycin/spectinomycin resistance and DsRed2 for selective tracking in soil microcosms. The *nasS* mutant of USDA110 was selected because it exhibits enhanced N₂O reductase activity compared with the wild type, as previously reported (29). Details regarding the construction and the two derivatives of each strain (SG09_1, SG09_3, USDA110_1, USDA110_2) as well as culture methods for maintenance and selective CFU enumeration are provided in the Supplementary Materials.

### Soil microcosm setup and inoculation

Bacterial cells grown on HM agar medium (30) were harvested, washed twice with phosphate-buffered saline, and resuspended in sterile distilled water to an OD660 of 1.0. Three replicate soil microcosms, each consisting of 10 g of soil in 50-mL tubes, were prepared for each combination of soil type (ANT, AT, or G) and inoculum treatment (inoculated or uninoculated control). A 500-μL aliquot of the bacterial suspension was inoculated into each microcosm. The microcosms were incubated in tightly sealed 50-mL tubes whose lids were wrapped with Parafilm to prevent moisture loss. The microcosms were incubated at 25°C in the dark. On Days 0, 14, 28, 57, and 249, from each microcosm, 1 g of soil was sampled, of which 0.5 g was used for viable counts and 0.5 g was stored at –80°C for DNA analyes.

### DNA extraction, library preparation, sequencing, and read preprocessing

DNA was extracted from 0.5 g of soil using the Extrap Soil DNA Kit Plus ver. 2 (BioDynamics Laboratory, Inc., Japan). For amplicon sequencing, the 16S rRNA gene was amplified using universal primers 515F/806R (31), and clade I *nosZ* genes (32) using the primers listed in Table S2. Indexed libraries were constructed according to the Illumina protocol. To reduce PCR/sequencing errors and correct for amplification biases, metabarcoding using amplicons with unique molecular identifiers (MAUI) -seq technology with 12-base unique molecular identifier (UMIs) was employed in the first PCR step (33). Libraries were sequenced on an Illumina MiSeq platform (600 cycles, paired-end). Raw reads were trimmed at a Phred score of 20 and merged using PEAR v0.9.6 (34). Unique sequences were dereplicated and quantified based on UMI counts following Bamba et al. (35). Sequences with fewer than 10 UMIs across all samples were discarded. Remaining sequences were clustered into OTUs using UCLUST (36) at a 1-base mismatch threshold. Although amplicon sequence variant (ASV) -level resolution was initially retained through UMI-based error correction, a small number of single-nucleotide variants consistently co-occurred with inoculant-derived sequences and exhibited identical temporal dynamics to those of the inoculant. These variants were therefore interpreted as residual sequencing errors rather than biologically meaningful ASV-level differences and were merged using a one-mismatch criterion. The centroid of each OTU was defined as the sequence with the highest total UMI count. 16S OTUs were taxonomically assigned using SINTAX (37) against the SILVA database (38) with a confidence threshold of 0.5. The *nosZ* OTUs were taxonomically assigned by BLASTn against a *nosZ* database (39), with identity and query coverage greater than 70%. Details on qPCR for 16S rRNA, *nosZ*, and *B. ottawaense*-specific genes are provided in the Supplementary Materials.

### Dimensional embedding, trajectory, and clustering analysis

Bray–Curtis and weighted UniFrac distance (40) matrices were calculated from the OTU table. Bray–Curtis distances were computed using the vegdist function from the vegan package v2.6 in R (41). For UniFrac calculations, a phylogenetic tree was constructed by aligning OTU sequences using the super5 command of Muscle5 v5.3 (42) with default parameters. The resulting alignment was then used to infer a maximum-likelihood tree with FastTree v2.1.11 (43), employing the generalized time-reversible (GTR) model and default settings. This tree was subsequently used to compute weighted UniFrac distances using the phyloseq package v1.50.0 (44).

Each distance matrix (Bray–Curtis and UniFrac) was used to generate ordination coordinates via four methods: PCoA (in the ape [45] package version 5.8.1), a widely used metric method that preserves pairwise Euclidean distances; Sammon mapping (46) (Sammon in the MASS package v7.3.64), rarely used in microbiome studies, yet valuable because it retains a metric framework while assigning greater weight to preserving small-scale distance relationships; non-metric multidimensional scaling (metaMDS in vegan), which preserves only the rank order of dissimilarities; and uniform manifold approximation and projection (UMAP, implemented in Python with umap-learn library v0.5.7), which optimizes both local neighborhoods and global topology (47). Embedding was performed at nine dimensionalities (d = 2, 3, 4, 7, 10, 15, 20, 30, and 70), and the resulting coordinates were exported for downstream analyses.

In addition to distance-based ordination, composition-based dimensional embeddings were generated using robust principal component analysis (RPCA) and phylogenetic RPCA implemented in the Gemelli v0.0.12 (48, 49). These methods apply a robust centered log-ratio (rCLR) transformation to count data, treating unobserved values as missing and thereby avoiding zero imputation. To ensure numerical stability, features with fewer than eight total counts across all samples were excluded prior to analysis (minimum feature count = 8). RPCA and phylogenetic RPCA embeddings were computed across a range of component numbers (d = 2, 3, 4, 7, 10, 15, 20, 30, and 70). For each dimensionality, sample coordinates were exported for downstream geometric analyses. In addition, distance matrices were reconstructed from the RPCA and phylogenetic RPCA embeddings and used for dimensionality-dependent PERMANOVA analyses.

To evaluate the directional similarity of microbial community trajectories over time, we computed the cosine of the angle (cosθ) between trajectory vectors derived from ordination coordinates. For each replicate, a trajectory vector was defined from Days 0 to 249. In addition, trajectory vectors were defined for each successive time interval (e.g., Days 0–14, 14–28, 28–57, and 57–249) to enable segment-level analyses. Pairwise cosθ values were calculated between all control and inoculated replicates for each soil. The resulting values were averaged to estimate the overall alignment of temporal dynamics between inoculum treatments. Calculations were performed using a custom Python script (Supplementary Materials). In addition to directional similarity, two complementary geometric properties of community trajectories were quantified: trajectory smoothness and linearity. Smoothness was calculated as the mean cosine similarity (cosθ) between successive trajectory segments defined by adjacent time intervals, quantifying local directional consistency along the trajectory. Linearity was calculated as the ratio of the Euclidean distance between the start (Day 0) and end (Day 249) points of a trajectory to the cumulative path length across intermediate time points, with higher values indicating straighter global trajectories.

To evaluate how dimensionality affects the balance between pattern resolution and homogenization, we computed three metrics: signal-to-noise ratio (SNR), mean k-nearest neighbor (kNN) distance with its coefficient of variation (CV), and the Hopkins statistic (50). SNR was defined as the ratio of between-treatment to within-treatment distances. Between-treatment distances were calculated between samples from the same soil and day but with different inoculum treatments. Within-treatment distances were calculated between samples sharing the same soil type, day, and treatment. The kNN metric used k = 5 and was computed using the Nearest Neighbors module from scikit-learn. The Hopkins statistic was computed using 50 random and 50 real points following Lawson and Jurs (50). All metrics were computed using a custom Python script (Supplementary Materials).

### Statistical analyses

All analyses were performed in R v4.2.2. CFU counts were compared by Welch’s t or Wilcoxon tests. Alpha diversity (Shannon, Chao1) was assessed with Wilcoxon or Dunn tests and Benjamini–Hochberg adjustment. Community composition was assessed by PERMANOVA on Bray–Curtis and weighted UniFrac matrices and RPCA-based distance using a global model (soil + day + inoculum treatment) as well as soil-specific models across pooled time points. Detailed descriptions of these analyzes are provided in the Supplementary Materials.

## RESULTS

### Long-term survival of *Bradyrhizobium* strains in three soils

**Fig 1:**
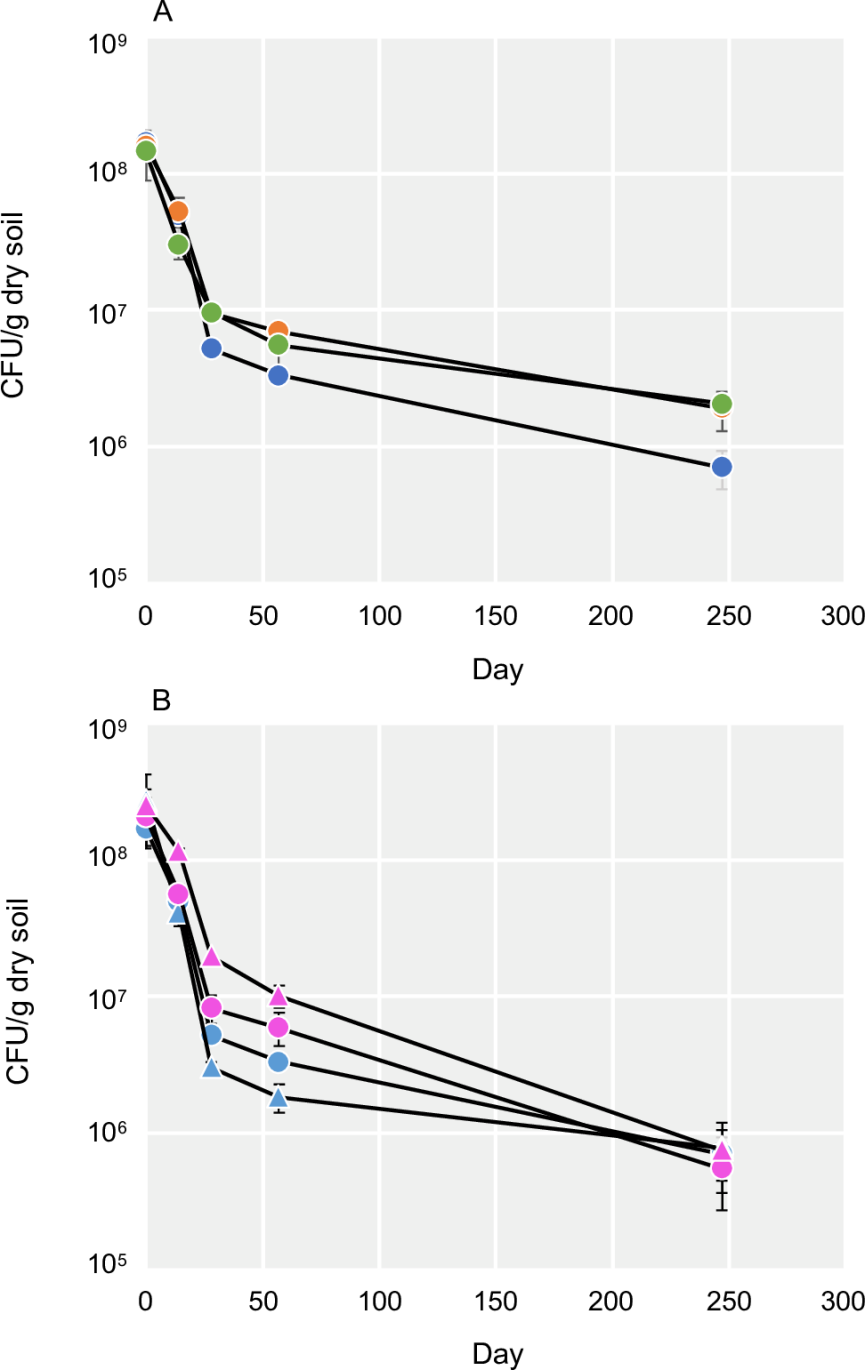
Selective tracking of viable *Bradyrhizobium* inoculants in soil microcosms over 249 days. (**A**) Temporal dynamics of CFUs for *B. ottawaense* SG09_1 inoculated into three soils: ANT (blue), AT (green), and G (orange). (**B**) Comparison of four inoculated strains in ANT: *B. ottawaense* SG09_1 (blue circle) and SG09_3 (blue triangle) or *B. diazoefficiens* USDA110_1 (pink circle) and USDA110_2 (pink triangle). CFUs were enumerated based on antibiotic resistance markers. Each data point represents the mean ± standard deviation (*n* = 3).

We monitored the viability of *Bradyrhizobium ottawaense* strain SG09_1 inoculated into three distinct soils—Andosol No Till (ANT), Andosol Till (AT), and Gleysol (G)—by tracking colony forming units (CFUs) over 249 days. After initially declining up to Day 28, CFU counts stabilized at 10⁵–10⁶ CFU/g in all soils (Fig. 1A). CFU counts in AT and G were approximately three-fold higher than those in ANT (*P* < 0.05). In ANT, qPCR-based quantification of *B. ottawaense* correlated well with CFU counts (r = 0.857, *P* < 0.001), demonstrating consistency between methods (Fig. S1A). As baseline data, 16S and *nosZ* gene copy numbers were measured by qPCR in control soils on Day 0. Across soils, 16S levels were similar (3.5–6.0 × 10⁹ copies/g), while *nosZ* abundance varied, with the lowest numbers being observed in AT (Fig. S1B). To examine whether survivability differed among strains, we compared four *Bradyrhizobium* strains (two marker-insertion variants each of SG09 and *B. diazoefficiens* USDA110) in ANT soil. Although USDA110 initially yielded higher CFU counts, differences among strains declined over time (Fig. 1B). By Day 249, all strains reached similar levels (≤1.6-fold difference, *P* > 0.05), indicating no significant differences in survival among the four tested strains.

### Whole-community analysis

#### Statistical analysis of inoculation effects on 16S-based community structure

Before examining community structure, we first assessed alpha diversity metrics. Inoculation with SG09_1 had no significant effect on Shannon diversity or Chao1 richness in any soil or at any time point (*P* > 0.05) (Fig. S2). In ANT soil, alpha diversity also remained unaffected across inoculum treatments (Control, SG09, and USDA110 derivatives) and all sampling days (*P* > 0.05). To assess the effects of inoculation on overall community structure, we conducted PERMANOVA using Bray–Curtis and weighted UniFrac distances. A global comparison incorporating soil type, time, and inoculum treatment showed that soil type explained the most variation, followed by time and treatment. The effects of inoculum treatment, although smaller, were statistically significant (Table 1), with UniFrac distances yielding slightly higher R² values than Bray–Curtis. To explore soil-specific responses, we performed soil-wise PERMANOVA by pooling samples across time. Inoculum treatment effects were strongest in G, moderate in AT, and not detectable in ANT. In ANT soil, no significant differences were detected among soils inoculated with the four *Bradyrhizobium* strains nor between any inoculated treatment and the non-inoculated control (*P* > 0.05).

**TABLE 1:** PERMANOVA results of whole community based on 16S rRNA and *nosZ* gene^*a*^

	Factor	16S rRNA amplicon				*nosZ* amplicon			
		Bray–Curtis		UniFrac		Bray–Curtis		UniFrac	
		R^2^	*P*-value	R^2^	*P*-value	R^2^	*P*-value	R^2^	*P*-value
Global	Treatment	0.009	0.015*	0.020	0.001*	0.182	0.001*	0.248	0.001*
	Soil	0.723	0.001*	0.727	0.001*	0.470	0.001*	0.530	0.001*
	Day	0.034	0.001*	0.083	0.001*	0.072	0.001*	0.054	0.001*
Soil-wise	ANT	0.034	0.346	0.038	0.277	0.288	0.001*	0.414	0.001*
	AT	0.086	0.002*	0.119	0.003*	0.500	0.001*	0.817	0.001*
	G	0.099	0.002*	0.179	0.003*	0.360	0.001*	0.531	0.001*

^*a*^ Asterisks indicate significance at *P* < 0.05.

#### Geometric analysis of 16S-based community dynamics across dimensions

**Fig 2:**
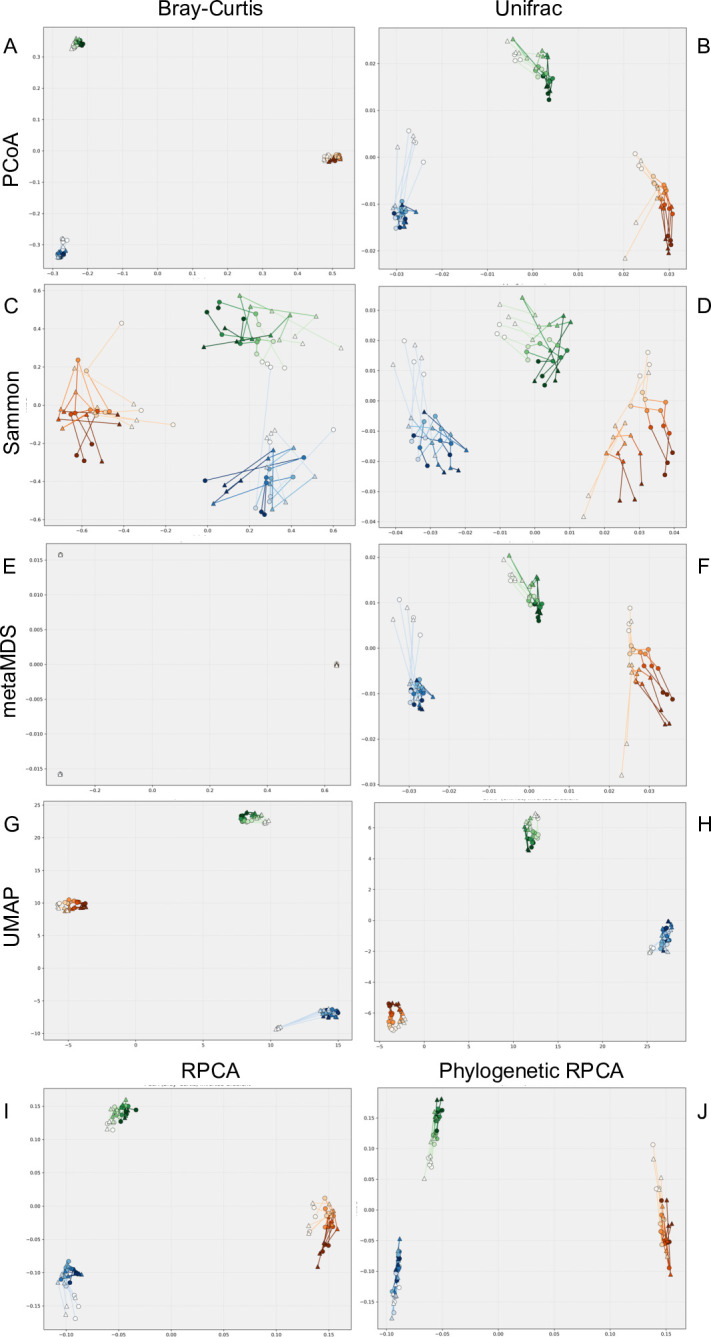
Visualization of inoculation-induced shifts in soil bacterial communities across time and soil types using multiple ordination methods. These ordination plots illustrate bacterial community structures based on 16S rRNA gene amplicon analysis. Panels **A–H** show ordination results derived from Bray–Curtis (**A, C, E, G**) and weighted UniFrac (**B, D, F, H**) distance matrices using PCoA (**A and B**), Sammon mapping (**C and D**), metaMDS (**E and F**), and UMAP (**G and H**). Panel **I** shows the two-dimensional embedding obtained by RPCA, and panel **J** shows the corresponding embedding obtained by phylogenetic RPCA, both based on rCLR-transformed count data. Circles represent uninoculated control soils, and triangles indicate SG09-inoculated soils. Lines connect samples from the same biological replicate across time points. Blue, green, and orange colors correspond to ANT, AT, and G soils, respectively. Within each soil, color shading indicates temporal progression from Days 0 (dark) to 249 (light).

To visualize variation in community structure by soil, time, and inoculum treatment, we performed two-dimensional ordination using Bray–Curtis and weighted UniFrac distances. In addition, we compared four dimensionality-reduction methods: (i) PCoA, (ii) Sammon mapping, (iii) metaMDS, and (iv) UMAP. With Bray–Curtis distances, PCoA, and metaMDS mainly grouped samples by soil type but lacked sufficient resolution to detect finer-scale variation (Fig. 2). Sammon mapping improved resolution of within-soil patterns, revealing clearer temporal and treatment effects. UMAP further enhanced separation between treatment groups. With weighted UniFrac distances, PCoA, Sammon mapping, and metaMDS produced similar ordination patterns and sharpened within-soil resolution compared with Bray–Curtis, because UniFrac’s incorporation of phylogenetic relatedness reduces the dissimilarity between soils. UMAP, by contrast, compressed local structure. To evaluate how each method partitioned variation between and within soils, we calculated β-dispersion for every ordination space. Sammon mapping best preserved the relative within-soil dispersion seen in the original UniFrac matrix (Table S3). Using composition-based embeddings rather than distance-based ordination, RPCA and phylogenetic RPCA primarily captured variation among soils (Fig. 2I and J). Across all visualizations, temporal progression within each soil was consistently observed, motivating further analysis of how inoculation affected the direction of these shifts.

**Fig 3:**
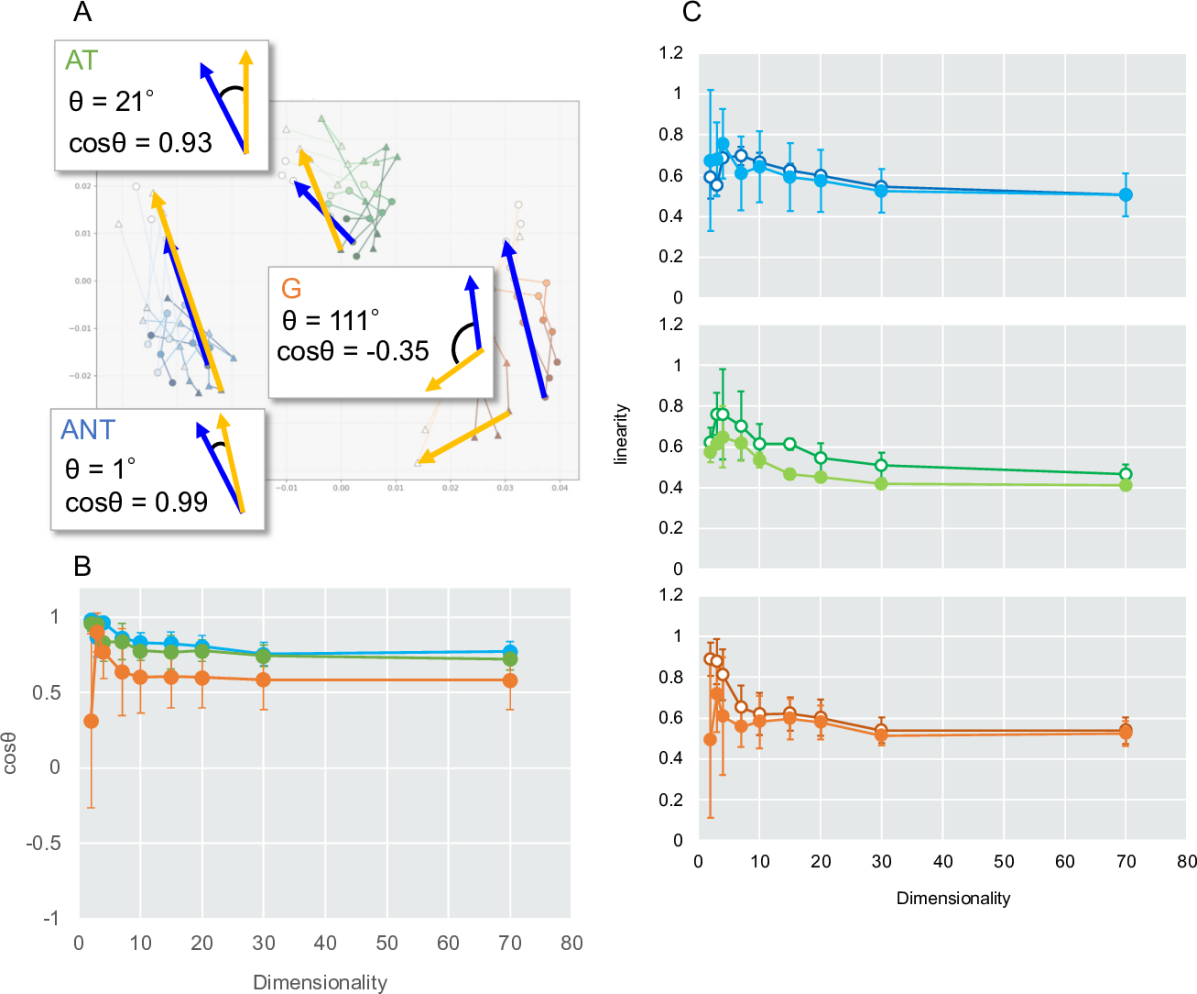
Impact of embedding dimensionality on geometric properties of 16S rRNA-based microbial community trajectories in weighted UniFrac–Sammon space. (**A**) Conceptual illustration of cosine similarity (cos θ) used to quantify directional alignment of community trajectories from Days 0 to 249 between control and inoculated soils. A value of 1.0 indicates identical orientation, 0 indicates orthogonality, and –1.0 indicates opposite orientations. The 2D mapping is the same as in Fig. 2D. (**B**) Mean pairwise cos θ values between control and inoculated soils across increasing embedding dimensions (d = 2–70). Error bars represent standard deviations. (**C**) Trajectory linearity across embedding dimensions, shown separately for each soil type (top: ANT; middle: AT; bottom: G). Linearity was calculated as the ratio of the Euclidean distance between the start (Day 0) and end (Day 249) points of a trajectory to the cumulative path length across time points, with higher values indicating straighter global trajectories. Control (open) and inoculated (closed) soils are shown for each soil. Error bars represent standard deviations.

To examine community trajectory directionality, we used two-dimensional coordinates from UniFrac-Sammon (Fig. 3A). For each soil, we calculated the cosine of the angle (cosθ) between the control and inoculated trajectories from Days 0 to 249. High cosθ values in ANT (0.98) and AT (0.96) indicated nearly parallel temporal shifts, while the low value in G (0.31) suggested a divergent response. To address distortion in low-dimensional space, we repeated the analysis in higher dimensions (d = 2, 3, 4, 7, 10, 15, 20, 30, 70). As dimensionality increased, cosθ values converged (to approximately 0.6–0.8, indicating broadly similar directions that were compressed in 2D (Fig. 3B). Segment-specific cosθ analysis revealed distinct temporal patterns among soils (Table S4). In ANT and AT soils, cosθ values were low during the early interval (Days 0–14) and higher during the late interval (Day 57–249), indicating greater directional alignment in later stages of the experiment. In contrast, G soil exhibited a gradual decrease in cosθ toward later intervals, suggesting increasing divergence in trajectory direction. These segment-level patterns are consistent with the high overall directional similarity observed for ANT and AT, and the comparatively divergent trajectory observed for G when cosθ was calculated between Days 0 and 249.

Trajectory smoothness analysis further characterized local directional dynamics (Fig. S3). Smoothness values showed substantial variability among replicates and did not increase monotonically with embedding dimensionality; in several cases, cosθ values decreased as dimensionality increased. Accordingly, changes in smoothness with increasing dimensionality were varied among soils and treatments. Notably, ANT control samples consistently exhibited low smoothness values (approximately −0.5 across dimensions), reflecting frequent changes in trajectory direction. By contrast, G soil showed higher smoothness values than ANT and AT across low to intermediate dimensions, indicating relatively smooth trajectories despite the overall divergence observed. We investigated how trajectory linearity changes with increasing dimensionality. Linearity was defined as the ratio of the Euclidean distance between Days 0 and 249 to its cumulative path length. Except for the control in G soil, trajectory linearity increased modestly at intermediate dimensionalities (approximately d = 3–7) and then gradually decreased with further increases in dimensionality (Fig. 3C).

We next compared these trajectory-based metrics across other ordination methods to evaluate how method choice influences directional and geometric interpretations (Fig. S4). For global trajectory directionality (Days 0–249 cosθ), PCoA and metaMDS showed patterns similar to those observed for Sammon, whereas UMAP yielded consistently high cosθ values (approximately 0.9) across dimensions and soils. Conversely, phylogenetic RPCA exhibited a pronounced decrease in cosθ with increasing dimensionality, reaching values near zero at d = 70. For trajectory smoothness, UniFrac-based methods (PCoA, Sammon, metaMDS, and UMAP) showed similar soil-dependent patterns, including consistently low smoothness in ANT controls and relatively higher smoothness in G across low to intermediate dimensions (Fig. S5). Under phylogenetic RPCA, these contrasts were attenuated. For trajectory linearity, a tendency toward local maxima at intermediate dimensionalities was observed not only in Sammon but also in PCoA and phylogenetic RPCA (Fig. S5).

**Fig 4:**
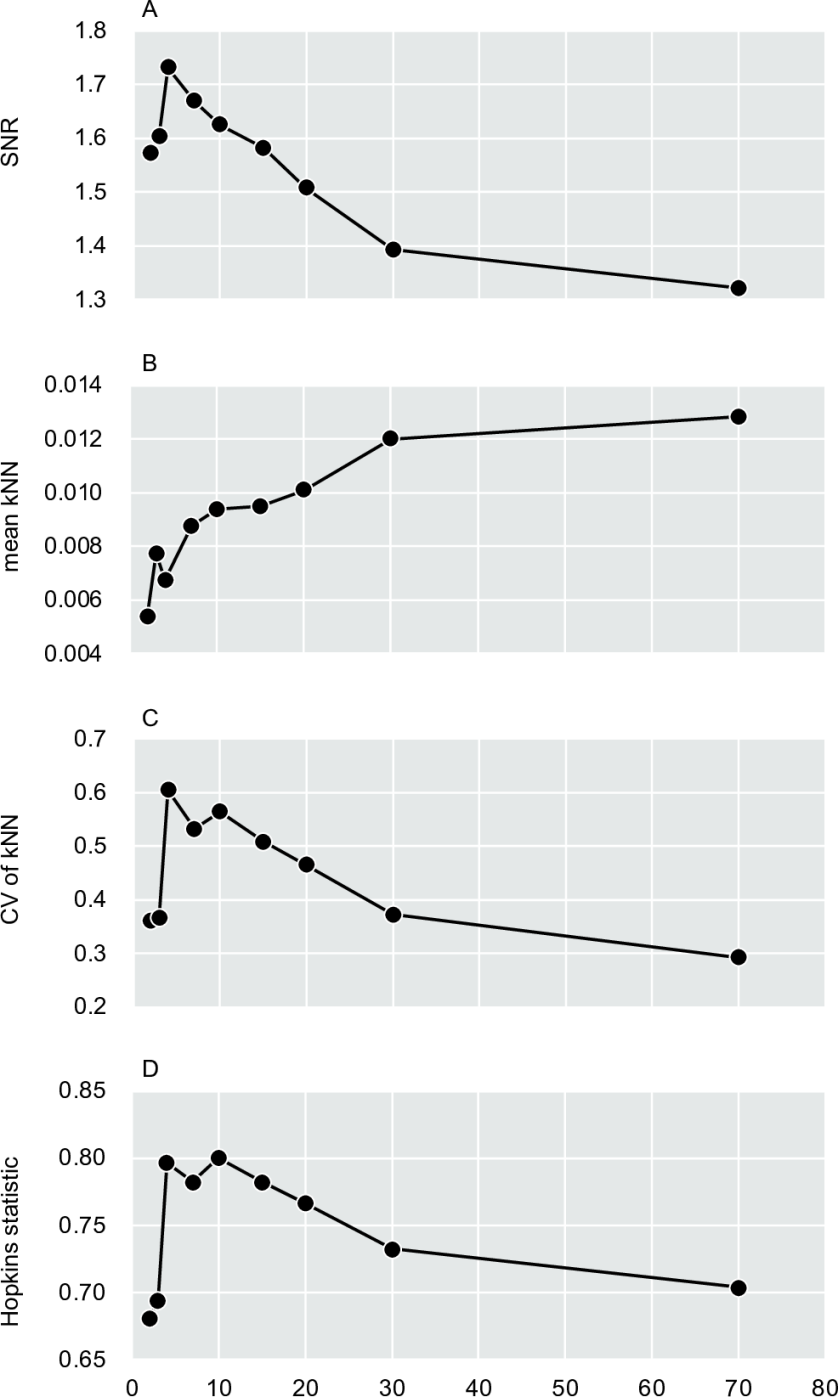
Dimensionality-dependent trends in metrics relevant to clustering across in Sammon-embedded space. (**A**) SNR, calculated as the ratio of between-treatment (signal) to within-treatment (noise) distances. (**B**) Mean kNN distance, representing the degree of local sample sparsity. (**C**) CV of kNN distances, indicating the degree of local spatial heterogeneity. (**D**) Hopkins statistic across dimensions. Values closer to 1 indicate stronger clustering, while values near 0.5 suggest random distribution of samples. All metrics were computed using coordinates from Sammon mapping of UniFrac distances across multiple dimensions (d = 2, 3, 4, 7, 10, 15, 20, 30, and 70).

Considering the finding of Beyer et al. (51) that nearest- and farthest-neighbor distances converge in high-dimensional space—an effect we hereafter refer to as distance homogenization—we evaluated quantitative indicators to examine how increasing dimensionality modulates the balance between structural resolution and distance homogenization. SNR (degree of inoculum-treatment-group separability), CV of kNN distances (degree of local heterogeneity), and the Hopkins statistic (degree of clustering tendency) peaked at around 4–10 dimensions (Fig. 4). In contrast, mean kNN distance (degree of local sparsity) showed an overall increasing trend with increasing dimensionality. Across other ordination methods, overall trends were broadly similar to those observed for Sammon, with the exception of UMAP; however, clear peaks in the CV of kNN distances and the Hopkins statistic were uniquely observed in Sammon-based embeddings (Fig. S6).

**Fig 5:**
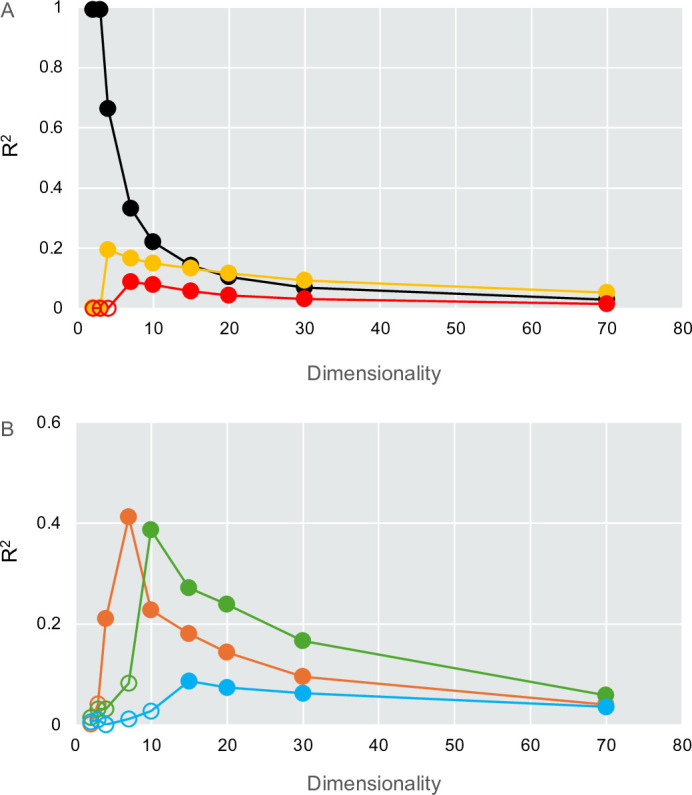
Dimension-dependent PERMANOVA results based on RPCA-derived distance matrices. PERMANOVA was performed using distance matrices reconstructed from RPCA embeddings across increasing dimensions (d = 2, 3, 4, 7, 10, 15, 20, 30, and 70). Panel **A** shows results from the global model, with the proportion of variance explained (R²) by soil (black), time (yellow), and inoculation treatment (red) plotted against dimensionality. Panel **B** shows soil-wise PERMANOVA results, with R² values for inoculation treatment shown separately for ANT (blue), AT (green), and G (orange) soils. Symbols indicate statistical significance (closed circles, *P* < 0.05; open circles, not significant).

We next examined how embedding dimensionality influences PERMANOVA results using distance matrices reconstructed from RPCA coordinates. Because RPCA outputs distance matrices derived from embeddings at each dimensionality, this framework allowed us to directly evaluate dimension-dependent changes in variance partitioning. At low dimensionalities, RPCA-based PERMANOVA was dominated by soil effects, whereas the contributions of time and inoculation treatment were minimal and often non-significant (Fig. 5A). As dimensionality increased, the explanatory power of time increased sharply around d = 4, and that of inoculation treatment increased around d = 7, at which point treatment effects also became statistically significant. Dimension-dependent peaks differed among soils in soil-wise PERMANOVA analyses (Fig. 5B), with inoculation effects emerging at lower dimensionalities in G (d = 7) than in AT (d = 10) and ANT (d = 15), indicating soil-specific differences in the dimensional scale at which inoculation effects become detectable. Similar trends were observed in phylogenetic RPCA-based analyses (Fig. S7).

#### Statistical analysis of inoculation effects on *nosZ*-harboring community structure

To evaluate the functional impact of inoculation, we analyzed the beta diversity of *nosZ*-harboring communities. A global comparison including soil type, time, and inoculum treatment revealed significant effects of all three factors (Table 1), with soil explaining the largest variation. In contrast to the 16S-based results, the effects of inoculant treatment on *nosZ* communities were consistently strong across all soils. Soil-wise comparisons confirmed these effects, with AT soil showing the greatest response—unlike the 16S-based results, where G exhibited the strongest response. Pairwise PERMANOVA revealed that all four strains significantly altered the *nosZ*-community structure compared with the control (*P* < 0.05 for all comparisons in Bray–Curtis; *P* < 0.05 in UniFrac except for 110_1, *P* = 0.051). Differences were observed between SG09 and USDA110 (*P* < 0.01) but not between their derivatives (*P* > 0.05).

### Sub-community analysis

#### Dynamics of 16S-based *Bradyrhizobium* communities

**Fig 6:**
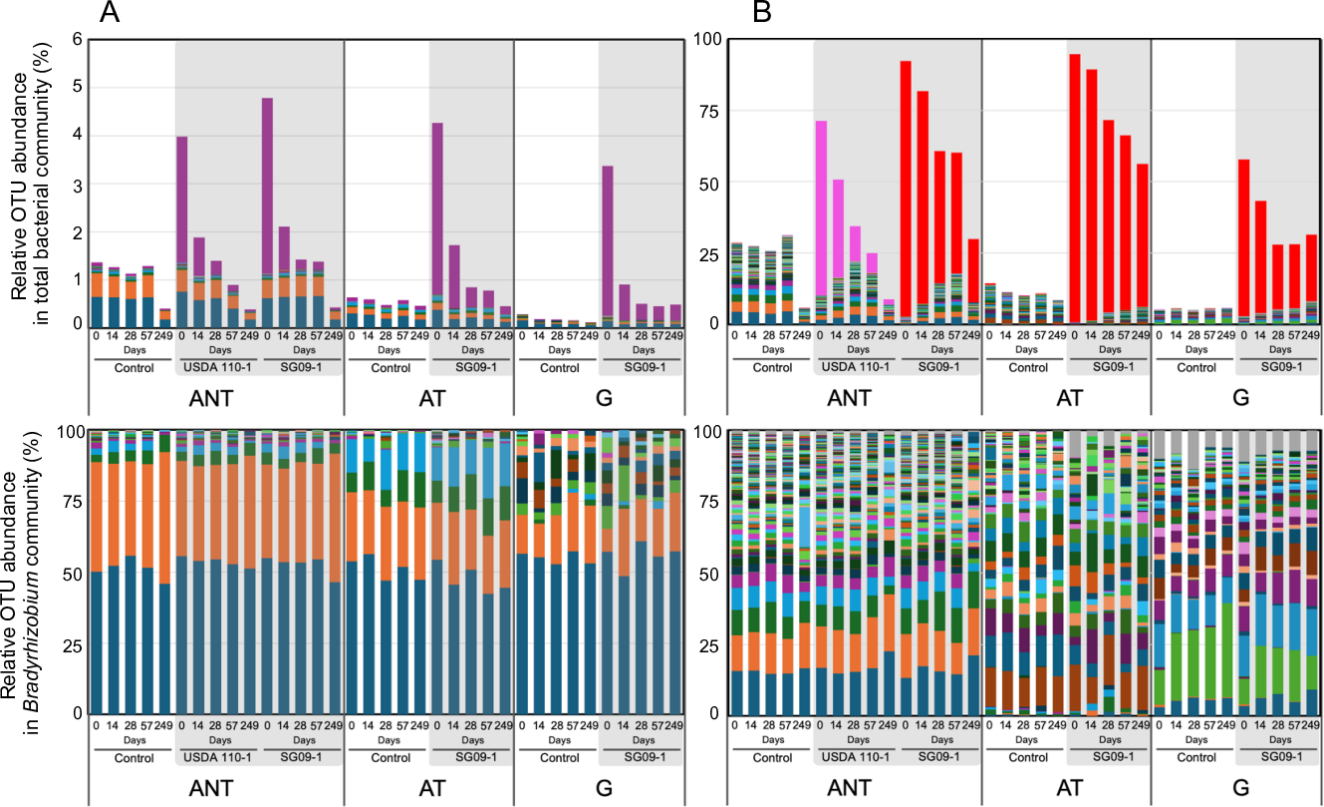
Time-series profiles of *Bradyrhizobium* OTU (clustered at 1-base mismatch threshold) compositions with and without inoculant OTUs in three soils. (**A**) 16S rRNA gene amplicon data. (**B**) *nosZ* gene amplicon data. For each panel, the top plots show the relative abundances of *Bradyrhizobium* OTUs as a proportion of the total bacterial community over time across three soils (ANT, AT, and G). The bottom plots display the normalized compositions of the remaining OTUs after removal of inoculant OTUs, rescaled to 100% to highlight shifts within the native *Bradyrhizobium* community. Colors represent individual OTUs and were assigned independently within each data set (16S rRNA and *nosZ*). In panel **A**, the inoculant OTU (shared by SG09 and USDA110) is shown in purple. In panel **B**, the SG09-derived OTU is shown in red, and the USDA110-derived OTU is shown in pink.

We examined the dynamics of *Bradyrhizobium* communities, comprising members of the same genus as the inoculated strains, to assess whether inoculation competitively displaced indigenous populations. The *Bradyrhizobium* communities in all three soils were dominated by the same two OTUs (clustered at a 1-base mismatch threshold), corresponding to phylogenetic branches including *B. elkanii* and *B. arachidis*–related lineages (Fig. S8). Inoculation caused a sharp increase in the relative abundance of inoculant OTUs corresponding to SG09 and USDA110 strains on Day 0 in all soils, followed by a gradual decline over time (Fig. 6A). On Day 249, these inoculant OTUs remained detectable in all inoculated soils, indicating long-term survival. Residual abundance varied among soils, with higher levels in G (0.33%), followed by AT (0.17%) and ANT (0.05%). PERMANOVA (Bray–Curtis and UniFrac) showed that inoculation significantly affected *Bradyrhizobium* community structure in all soils (*P* < 0.05), including ANT, where no effect of inoculation was seen at the whole-community level. Additionally, no substantial differences were observed among the four strains in ANT (*P* > 0.05).

#### Dynamics of *nosZ*-harboring *Bradyrhizobium* communities

**Fig 7:**
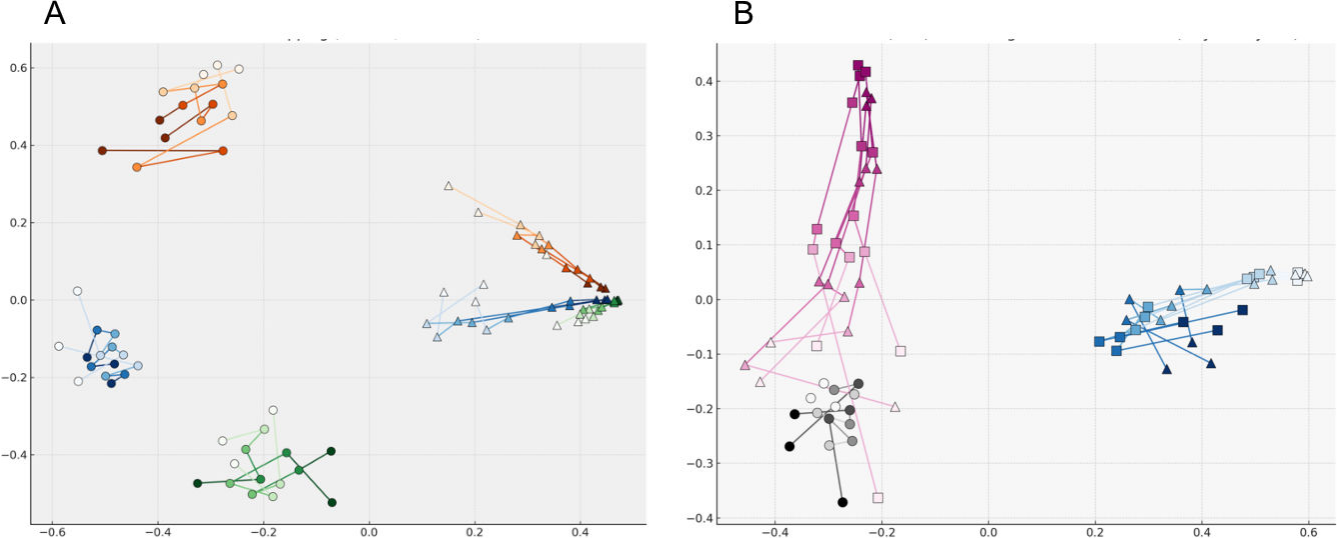
Visualization of *Bradyrhizobium* community responses to inoculation based on *nosZ* amplicon data using Sammon-embedded space based on weighted UniFrac distances. (**A**) Community structures of SG09_1-inoculated (triangles) and control (circles) in ANT (blue), AT (green), and G (orange) soils, with temporal progression indicated by change in shade from dark (Day 0) to light (Day 249). (**B**) Comparison of community shifts in ANT soil following inoculation with SG09 derivatives (blue) and USDA110 derivatives (pink). Symbols indicate SG09_1 and USDA110_1 (triangles), SG09_3 and USDA110_2 (squares), and uninoculated controls (gray circles). Only OTUs assigned to the genus *Bradyrhizobium* were included in the analysis.

On Day 0, the relative abundance of all *nosZ* OTUs annotated as *Bradyrhizobium* in control soils ranged from 5% to 29% (Fig. 6B)—considerably higher than the relative abundance of *Bradyrhizobium* estimated with the 16S data set. The OTU compositions in the different soils were highly diverse, with distinct dominant OTUs being observed in each soil (Fig. S8B). *NosZ*-harboring *Bradyrhizobium* communities in inoculated soils differed significantly from those in non-inoculated controls across all three soils, as evidenced by PERMANOVA (Table 2) and UniFrac-based two-dimensional ordination (Fig. 7A). However, when the inoculant OTU was excluded, the composition of the remaining native *Bradyrhizobium* OTUs appeared largely unchanged from the control (Fig. 6B bottom). A similar trend was also observed in the 16S data set (Fig. 6A bottom). To assess the contribution of the SG09-derived OTU to the observed PERMANOVA variance, we reanalyzed the data set after removing the SG09-derived OTU. This exclusion markedly reduced the explanatory power of the inoculum treatment (comparisons between inoculated and non-inoculated control soils) in PERMANOVA tests. Although significant differences remained in some comparisons (q < 0.05), the R² value dropped by approximately 90% compared to the full data set (Table 2). This reduction indicates that most of the variance detected in distance-based analyses was largely accounted for by the presence of the SG09-derived OTU, while the structure of the remaining indigenous *Bradyrhizobium* community showed limited divergence from the non-inoculated control.

**TABLE 2:** Effect of SG09-derived OTU on PERMANOVA results of the *nosZ*-based *Bradyrhizobium* community^*a*^^,^^*b*^^,^^*c*^

Metric	Soil	Data set	R^2^	*P*-value
Bray	ANT	All OTU	0.794	0.001*
		Without SG09 OTU	0.035	0.424
	AT	All OTU	0.830	0.001*
		Without SG09 OTU	0.063	0.009*
	G	All OTU	0.826	0.001*
		Without SG09 OTU	0.119	0.003*
UniFrac	ANT	All OTU	0.917	0.001*
		Without SG09 OTU	0.052	0.171
	AT	All OTU	0.936	0.001*
		Without SG09 OTU	0.065	0.066
	G	All OTU	0.899	0.001*
		Without SG09 OTU	0.201	0.003*

^*a*^ OTUs were clustered at a 1-base mismatch threshold.

^*b*^ PERMANOVA was conducted using only those OTUs taxonomically assigned to *Bradyrhizobium* based on *nosZ* gene amplicon data.

^*c*^ Asterisks indicate significance at *P* < 0.05.

Although the 16S rRNA analysis failed to distinguish SG09 and USDA110, the *nosZ* analysis successfully separated the inoculated strains based on their unique sequence signatures (Fig. 6B). In ANT, the survivability of SG09 strains was high (approximately 21%), whereas that of USDA110 strains was low (approximately 1.9%). The positions of USDA110-inoculated samples gradually shifted toward those of the uninoculated control over time (Fig. 7B).

## DISCUSSION

### Microcosm approach for long-term tracking

Previous studies have reported mixed results regarding the recovery of inoculated strains under field conditions: some were able to recover rhizobial inoculants even years after application (13), whereas others failed to detect inoculant-derived sequences (15). These contrasting outcomes highlight the difficulty of reliably tracking inoculated strains in the field due to dispersal, migration, and environmental heterogeneity (16–19). In contrast, our microcosm approach ensured containment and sensitive detection. By using *Bradyrhizobium* strains tagged with antibiotic resistance, we were able to selectively track viable inoculants even in soils with rich native *Bradyrhizobium* communities. This enabled us to directly link recovered cells to specific OTUs identified by 16S and *nosZ* amplicon sequencing, facilitating high-resolution tracking of inoculated strains across taxonomic and functional contexts. At the same time, the early dominance of the inoculated strain may have reduced the detection sensitivity for low-abundance OTUs; therefore, our interpretation is confined to community members that were detectable under the present sequencing depth.

### Ordination as an analytical framework in microbial ecology

Previous studies have demonstrated that the choice of distance metric can influence both visualization and statistical interpretation (52, 53). Initially, we expected that Bray–Curtis would more sensitively detect OTU-level differences. However, substantial background noise in the control soils reduced the ability to detect the effect of inoculated OTUs. UniFrac, by down-weighting shallow phylogenetic turnovers, more effectively highlighted the influence of the inoculated strains. This underscores the value of combining both metrics to capture subtle and complex community dynamics resulting from microbial inoculation.

PCoA and metaMDS with Bray–Curtis dissimilarity are widely used in microbial ecology (52, 54); however, we found that Bray–Curtis collapsed the local variation in our data set due to large differences between soils. In contrast, Sammon mapping resolved within‐soil variation and highlighted temporal trajectories and inoculation effects. UMAP—which is a relatively recent addition to microbial ecology (55)—yielded an intermediate ordination between metaMDS and Sammon, reflecting its balance of local and global structure. It also produced consistently high cos θ values (approximately 0.9) across dimensions and soils, indicating directional stability; however, this uniformity failed to capture the divergence unique to G, which was clearly observed in the UniFrac–Sammon space. In our study, we prioritized the use of Sammon mapping to more accurately capture subtle inoculation effects—despite its lower stability—due to its higher sensitivity to local structural differences.

While overall trajectory direction provides a global measure of community change, direction alone does not capture local geometric properties of trajectories. We therefore evaluated trajectory smoothness and linearity to characterize local directional stability and path straightness. Trajectory smoothness showed substantial variability among soils and replicates and did not change monotonically with increasing dimensionality. Similarly, trajectory linearity tended to peak at intermediate dimensions (approximately d = 3–7) before declining at higher dimensions. Consistent with this pattern, similar intermediate-dimensional peaks were also observed in clustering-related metrics, including SNR and the Hopkins statistic, suggesting a shared dimensional scale at which trajectory geometry and clustering structure are most strongly resolved. Collectively, these results suggest that higher dimensionality does not necessarily improve biological resolution and that an intermediate dimension may offer the best balance between homogenization and fidelity. These findings underscore the importance of dimensional selection when using ordination outputs for downstream geometric analysis, particularly in microbiome studies where both local sensitivity and global coherence are needed.

Building on this dimensional perspective, RPCA and phylogenetic RPCA provided a complementary analytical framework by explicitly linking ordination dimensionality to statistical inference. In these analyses, soil effects dominated at low dimensionalities, whereas time and inoculation effects emerged only at intermediate dimensions, with the dimensional scale at which treatment effects became detectable differing among soils. These results reinforce the idea that the detectability of inoculation effects is inherently dimension-dependent and highlight RPCA-based analyses as a useful bridge between ordination geometry and hypothesis-driven statistical testing.

The stronger inoculation signal in our *nosZ*-based analyses reflects both differences in abundance and taxonomic specificity. Because *nosZ* copy numbers (approximately 10^6^ g^−^¹) are three orders of magnitude lower than those of 16S (approximately 10^9^ g^−^¹), sequencing targets a much narrower functional guild dominated by *Bradyrhizobium*, amplifying treatment effects. We saw the same “scope-narrowing boost” when shifting from whole communities to the 16S-based *Bradyrhizobium* subset. An important consideration when comparing *Bradyrhizobium* abundances inferred from 16S rRNA and *nosZ* data sets is that *nosZ* is unevenly distributed within the genus (56). In uninoculated soils, the dominant 16S-based OTUs were affiliated with *B. elkanii*-related lineages (Fig. S8A), which largely lacks *nosZ*, rendering these populations undetectable in *nosZ*-based analyses. As a result, the two data sets represent overlapping but non-identical subsets of the *Bradyrhizobium* community.

### Ecological context of inoculant survivability and community impact

In the present study, soils differed in several measured physicochemical properties, including total nitrogen, extractable inorganic nitrogen, pH, and iron content (Table S1). However, no single parameter showed a monotonic relationship with inoculation outcomes across the three soils, indicating that bulk soil properties alone cannot fully explain the observed differences.

We therefore examined biological and community-level contexts that may constrain inoculation outcomes. Using *nosZ*-based UniFrac distances at Day 0, the native ANT community showed the smallest distance to SG09 (0.225), followed by AT (0.303) and G (0.346); whereas USDA110 was slightly closer to the native ANT community than was SG09 (0.217 vs. 0.225). Consistent with this pattern, inoculation effects were smallest in ANT and particularly limited for USDA110.

Although total bacterial abundance (16S qPCR) was similar across soils, the relative abundance of *Bradyrhizobium* (16S amplicon) was highest in ANT, followed by AT and G. In contrast, inoculation effects were strongest in G, weaker in AT, and minimal in ANT. The inverse trend was also observed for *nosZ*: qPCR showed the lowest *nosZ* gene copy numbers in AT, yet community shifts were most pronounced in AT, followed by G and then ANT. Although this evaluation is based on a limited number of soils, these observations are consistent with the concept that phylogenetic similarity and the initial abundance of indigenous populations may influence inoculation outcomes. Rather than identifying definitive drivers, they suggest that such factors modulate how inoculation effects are detected. Notably, their influence became apparent only within specific analytical spaces and dimensional scales, highlighting the importance of analytical dimensionality in quantitatively revealing soil-dependent inoculation effects.

### Conclusions and implications

This study successfully tracked the long-term survival and ecological impacts of *Bradyrhizobium* inoculation across three distinct soils under controlled microcosm conditions. We found that while survival varied by type of soil and strain, inoculated strains persisted in all soils without causing drastic shifts in the native community structures. Notably, the inoculated strains maintained dominance (20%–40%) within *nosZ*-harboring communities even after 249 days. These findings demonstrate the potential of microbial bioaugmentation to enhance functional potential without disturbing indigenous microbial communities, offering valuable perspectives for sustainable agricultural and environmental applications.

In contrast to the conventional use of dimensionality reduction as a visualization tool, our study reinterprets ordination as a high-dimensional embedding framework for subsequent geometric analysis. This use of ordination space as an embedding framework beyond conventional 2D or 3D visualization remains rare in microbial ecology. Armstrong et al. (52) emphasized the importance of distinguishing visualization from multivariate analysis, noting that although dimensionality reduction is often used for exploratory overview by human observers in 2D or 3D, the intrinsic dimensionality of microbiome data may be higher (52). Our study serves as one example of the potential application of dimensionality reduction to quantitatively assess microbial community dynamics. This approach allowed us to detect subtle but biologically meaningful patterns—particularly in soil-specific responses to inoculation—that would otherwise be difficult to discern. Our findings illustrate how dimensionality reduction can serve not only as an interpretive aid, but also as an analytical framework for hypothesis testing in microbial ecology, and how it may help clarify inoculation effects in field settings, where microbial dynamics is often masked by background variability.

## Data Availability

The amplicon reads generated in this study have been deposited in the DDBJ Sequence Read Archive under BioProject accession PRJDB35655, with 16S rRNA reads available under accession numbers DRR719074–DRR719208 and clade I *nosZ* reads under DRR719209–DRR719343.

## SUPPLEMENTAL MATERIAL

Supplemental material spectrum.02880-25-s0001.pdf Supplemental methods, Tables S1 to S4, Fig. S1 to S8, and Python scripts for computing cos θ, SNR, kNN, and Hopkins statistic.

ASM does not own the copyrights to Supplemental Material that may be linked to, or accessed through, an article. The authors have granted ASM a non-exclusive, world-wide license to publish the Supplemental Material files. Please contact the corresponding author directly for reuse.
