# Supplementary material for: Higher-dimensional ordination analysis teases out impacts of Bradyrhizobium bioaugmentation on native soil microbial communities
Source: Microbiol Spectr. 2026 Feb 18;14(4):e02880-25. doi: 10.1128/spectrum.02880-25 (PMC13055284; doi:10.1128/spectrum.02880-25)
Supplement: Supplemental material — Supplemental methods, Tables S1 to S4, Fig. S1 to S8, and Python scripts for computing cos θ, SNR, kNN, and Hopkins statistic. [file spectrum.02880-25-s0001.pdf]

Supplementary Materials

1. **Supplementary methods**
2. **Supplementary tables (Table S1-S4)**
3. **Supplementary figures (Fig. S1-S8)**
4. **Python scripts for computing cos θ, SNR, kNN, and Hopkins statistic**

1. Supplementary methods

1-1. Constriction and cultivation bacterial strains

As Bradyrhizobium strains with high N_2O-reducing activity, *Bradyrhizobium ottawaense* SG09 and a *nasS* mutant of *B. diazoefficiens* USDA110 (1) were used in the experiment. To enable selective tracking in soil, these strains were labeled with antibiotic resistance and fluorescence by using the pUT-based mini-Tn5 vector pBjGroEL4::dsRed2, which carries a streptomycin/spectinomycin resistance gene and the *DsRed2* gene under the control of the constitutive *BjGroEL4* promoter (2) (3). Two insertion variants were used for each strain, differing in the genomic integration site of the marker: SG09_1 (inserted at RS07515), SG09_3 (at RS22330), USDA110_1 (at *blr6172*), and USDA110_2 (at *blr4679*). These strains were cultivated on HM salt medium (4) supplemented with 0.1% (w/v) arabinose and 0.025% (w/v) yeast extract.

1-2. Selective plating and CFU enumeration

To quantify culturable cells of the inoculated strains, 0.5 g of soil was suspended in 4.5 ml of PBS.

Serial dilutions were plated on HM agar (4) containing antibiotics: 100 μg ml^{-1} streptomycin and polymyxin B, 50 μg ml^{-1} chloramphenicol, 2 μg ml^{-1} crystal violet, and 200 μg ml^{-1} cycloheximide. After 10 days of incubation at 30°C in the dark, colonies were counted to determine CFU. The inoculant strains harbored a chromosomally integrated *DsRed* marker gene, giving a faint pink colony coloration that enabled visual discrimination.

1-3. Library preparation for amplicon sequencings

DNA was extracted from 0.5 g of soil using the Extrap Soil DNA Kit Plus ver. 2 (BioDynamics Laboratory Inc., Japan). The 16S rRNA gene was amplified using the universal primers 515F and 806R (5), and clade I *nosZ* genes were amplified using specific primers (6) (see Table S2). PCR was performed with KAPA HiFi HotStart ReadyMix (KAPA Biosystems). The first round of amplification consisted of 25 cycles for 16S rRNA (annealing at 55°C for 30 sec) and 35 cycles for *nosZ* (annealing at 53°C for 30 sec). Indexing was performed in a second PCR step using the Nextera XT Index Kit v2 (Illumina). The resulting libraries were sequenced with 300 bp paired-end reads on an Illumina MiSeq platform using the MiSeq Reagent Kit v3 (Illumina).

1-3. Quantification of target genes in soil samples

For quantitative PCR targeting the 16S rRNA gene, nosZ, and *Bradyrhizobium ottawaense*-specific genes, 0.5 g of soil was spiked with 3.9×10^7 copies of the pEGFP plasmid (Clontech, USA) prior to DNA extraction, serving as an internal standard to estimate DNA extraction efficiency (7). The extracted DNA was diluted 10-fold and used as the template for qPCR, which was performed on a

LightCycler 96 system (Roche) using FastStart Essential DNA Green Master Mix (Roche) and gene-specific primers (Table S2). The qPCR was run for 40 cycles with an annealing temperature of 60°C for 10 sec. The absolute abundance of each target gene (copies per gram of dry soil) was calculated by correcting the qPCR results using DNA extraction efficiency estimated from the recovery rate of spiked pEGFP.

To enable quantitative PCR detection of *B. ottawaense* inoculants in soil, we designed a species-specific primer set using a comparative genomics approach. Genome sequences from *B. ottawaense* strains SG09 and OO99^{T}, along with related reference strains (*B. diazoefficiens* USDA110^{T}, USDA122, NK6, and XF7; *B. japonicum* USDA6^{T}, J5, and SEMIA5079; and *B. elkanii* USDA61), were analyzed using the EDGAR 3.0 (8) web-based platform for pan-genome and core-genome analyses. A total of 271 genes unique to *B. ottawaense* were initially identified. Genes with sequence similarity to non-*ottawaense* species were excluded based on NCBI-BLAST searches. The specificity of candidate genes was further evaluated using genomic DNA from the reference strains and soil extracted DNA. As a result, a gene corresponding to locus RS09935 in strain SG09 was selected as the specific marker for *B. ottawaense*.

1-4. Statistical analysis

Statistical analyses were performed in R version 4.2.2 unless otherwise noted. Differences in alpha diversity metrics (Shannon index and Chao1 richness, calculated using the vegan) between groups were assessed using the Wilcoxon rank-sum test. For multiple-group comparisons, Dunn’s test was

used with Benjamini–Hochberg correction. Correlation between CFU and qPCR-based estimates was evaluated using Pearson's correlation. To assess community-level differences in microbial composition, PERMANOVA was performed using the adonis2() function from the vegan package, based on both Bray–Curtis and weighted UniFrac distances. Two modeling strategies were used: (I) a global model including soil type, sampling day, and inoculum treatment; (II) soil-wise treatment effects pooling all time points. Pairwise PERMANOVA tests (PairwiseAdonis package v0.4.1) were conducted to compare each inoculated group with the control and among inoculant strains. P-values were adjusted using the Benjamini–Hochberg method. Depending on the variance structure, either Welch's t-test or Wilcoxon rank-sum test was used to compare CFU counts across conditions.

References

1. Sánchez C, Itakura M, Okubo T, Matsumoto T, Yoshikawa H, Gotoh A, Hidaka M, Uchida T, Minamisawa K. 2014. The nitrate-sensing NasST system regulates nitrous oxide reductase and periplasmic nitrate reductase in. Environmental Microbiology 16:3263-3274.
2. Hayashi M, Shiro S, Kanamori H, Mori-Hosokawa S, Sasaki-Yamagata H, Sayama T, Nishioka M, Takahashi M, Ishimoto M, Katayose Y, Kaga A, Harada K, Kouchi H, Saeki Y, Umehara Y. 2014. A Thaumatin-Like Protein, Rj4, Controls Nodule Symbiotic Specificity in Soybean. Plant and Cell Physiology 55:1679-1689.

3. Okubo T, Fukushima S, Itakura M, Oshima K, Longtonglang A, Teaumroong N, Mitsui H, Hattori M, Hattori R, Hattori T, Minamisawa K. 2013. Agromonas oligotrophica (*Bradyrhizobium oligotrophicum*) is a nitrogen-fixing symbiont of *Aeschynomene* indica. Applied and Environmental Microbiology 79:2542-2551.

4. Cole MA, Elkan GH. 1973. Transmissible Resistance to Penicillin-G, Neomycin, and Chloramphenicol in Rhizobium-Japonicum. Antimicrobial Agents and Chemotherapy 4:248-253.

5. Klindworth A, Pruesse E, Schweer T, Peplies J, Quast C, Horn M, Glöckner FO. 2013. Evaluation of general 16S ribosomal RNA gene PCR primers for classical and next-generation sequencing-based diversity studies. Nucleic Acids Research 41: 248-253.

6. Zhang B, Penton CR, Yu Z, Xue C, Chen Q, Chen Z, Yan C, Zhang Q, Zhao M, Quensen JF, Tiedje JM. 2021. A new primer set for Clade I nosZ that recovers genes from a broader range of taxa. Biology and Fertility of Soils 57:523-531.

7. Park JW, Crowley DE. 2005. Normalization of soil DNA extraction for accurate quantification real-time PCR and of target genes by DGGE. Biotechniques 38:579-586.

8. Dieckmann MA, Beyvers S, Nkouamedjo-Fankep RC, Hanel PHG, Jelonek L, Blom J, Goesmann A. 2021. EDGAR3.0: comparative genomics and phylogenomics on a scalable infrastructure. Nucleic Acids Research 49:W185-W192.

2. Supplementary tables

Table S1. Properties of soils used in this study

	ANT	AT	G
Soil Type	Andosol (no-tillage)	Andosol (tillage)	Gleysol
Total organic carbon(%)	11.0	5.1	3.0
Total nitrogen (%)	0.59 (ref1)	0.41 (ref1)	0.19 (ref3)
KCl extractable inorganic N (mg kg^{-1})	69-75 (ref2)	43-77 (ref2)	NR
Water-extractable NO3$^-$ (μg/g)	29.3 (ref1)	5.9 (ref1)	NR
Phosphate-buffer-extractable NO3$^-$ (μg/g)	27.9 (ref1)	6.2 (ref1)	NR
pH (H2O)	6.53	6.53	6.80
Soil Fe*(%)	3.5	4.5	1.3

*Soil Fe represents pedogenic Fe content and measured by sequential extraction of pyrophosphate, acid oxalate, and dithionite-citrate reagent following Wagai et al. (2020).

ref1) Data from Wagai, et al. (2013) Global Change Biology 19: 1114-1125.

ref2) Data from Shingubara, et al. (2025) Geochemical Journal 59: 144-162.

ref3) Data from Hamamoto et al. (2025) Paddy and Water Environment 23: 333-342.

NR, not reported in the cited literature.

Table S2. Primer sets for amplicon sequencings and quantitative PCR.

Experiment	Target gene	Primer name	Primer sequence (5' to 3')	References
Amplicon sequencing	16S rRNA V3-V4	16S_341F_MAUI_illumina	*TCGTCGGCAGCGTCAGATGTGTATAAGAGACAG* **NNNHNNNWNNNHCCTACGGGNGGCWGCAG**	Klindworth et al., 2013
		16S_785R_illumina	*GTCTCGTGGGCTCGGAGATGTGTATAAGAGACAG* **GACTACHVGGGTATCTAATCC**	
	nosZ clade I	nosZ_F_MAUI_illumina	*TCGTCGGCAGCGTCAGATGTGTATAAGAGACAG* **NNNHNNNWNNNHGGCAARCTVTCDCCVAC**	Zhang et al., 2021
		nosZ_R_illumina	*GTCTCGTGGGCTCGGAGATGTGTATAAGAGACAG* **AVCGGTCYTTVGAGAAYTT**	
Quantitative PCR	16S rRNA V3-V4	16S_341F	CCTACGGGNGGCWGCAG	Klindworth et al., 2013
		16S_785R	GACTACHVGGGTATCTAATCC	
	nosZ clade I	nosZ2F	CGGRACGGCAASAAGGTSMSSGT	Henry et al., 2006
		nosZ2R	CAKRTGCAKSGCRTGGCAGAA	
	B. ottawaense	RS09935_F	TCCTGCTGACCTTGAATCGT	in this study
		RS09935_R	CACCGTCGTTCAACCAGTTT	
	pEGFP-N1	960F	CCAGGAGCGCACCATCTT	Park et al., 2005
		1070R	AAGTCGATGCCCTTCAGCT	

PCR reactions were performed using approximately 5 ng of template DNA. Primer concentrations were 1.0 μM for 16S rRNA gene amplification and 2.5 μM for clade I nosZ amplification.

Klindworth A, Pruesse E, Schweer T, Peplies J, Quast C, Horn M, Glöckner FO. 2013. Evaluation of general 16S ribosomal RNA gene PCR primers for classical and next-generation sequencing-based diversity studies. Nucleic Acids Research 41.

Zhang B, Penton CR, Yu Z, Xue C, Chen Q, Chen Z, Yan C, Zhang Q, Zhao M, Quensen JF, Tiedje JM. 2021. A new primer set for Clade I nosZ that recovers genes from a broader range of taxa. Biology and Fertility of Soils 57:523-531.

Henry S, Bru D, Stres B, Hallet S, Philippot L. 2006. Quantitative detection of the nosZ gene, encoding nitrous oxide reductase, and comparison of the abundances of 16S rRNA, narG, nirK, and nosZ genes in soils. Applied and Environmental Microbiology 72:5181-5189.

Park JW, Crowley DE. 2005. Normalization of soil DNA extraction for accurate quantification real-time PCR and of target genes by DGGE. Biotechniques 38:579-586.

Table S3. Comparison of global and within-soil dispersion across ordination methods and the original Unifrac distance matrix

	PCoA*	Sammon*	metaMDS*	UMAP*	Original distance**
Global	0.027	0.030	0.024	12.5	0.040
ANT	0.0052	0.011	0.0052	0.87	0.019
AT	0.0038	0.009	0.0038	0.80	0.017
G	0.0055	0.013	0.0055	0.77	0.024
ANT/Global	0.20	0.38	0.22	0.070	0.48
AT/Global	0.14	0.30	0.16	0.064	0.41
G/Global	0.21	0.45	0.23	0.062	0.59

*) β-dispersion—defined as the mean Euclidean distance from each sample to its centroid—was computed from the ordination coordinates: a global value was obtained from all samples, whereas within-soil values were calculated separately for each soil using all time points and treatments.

**) The original Unifrac values represent mean pairwise distances within each soil and across all samples.

Table S4. Segment-specific cosine similarity (cos θ) between successive trajectory vectors across embedding dimensions

		Dimensionality								
Soil	Day segment	2	3	4	7	10	15	20	30	70
ANT	0-14	0.06 (0.93)	0.08 (0.85)	-0.09 (0.55)	-0.05 (0.46)	0.02 (0.5)	0.02 (0.4)	0.02 (0.38)	0.02 (0.28)	0.02 (0.22)
	14-28	0.85 (0.19)	0.68 (0.28)	0.74 (0.29)	0.63 (0.19)	0.59 (0.19)	0.59 (0.17)	0.48 (0.12)	0.38 (0.17)	0.24 (0.11)
	28-57	0.32 (0.62)	0.08 (0.45)	-0.04 (0.76)	-0.16 (0.42)	-0.04 (0.38)	0.08 (0.38)	0.11 (0.29)	0.1 (0.21)	0.07 (0.16)
	57-249	0.97 (0.02)	0.89 (0.09)	0.94 (0.04)	0.89 (0.06)	0.87 (0.07)	0.86 (0.08)	0.85 (0.07)	0.78 (0.08)	0.78 (0.06)
AT	0-14	0.58 (0.43)	0.61 (0.35)	0.3 (0.71)	0.37 (0.56)	0.38 (0.46)	0.27 (0.39)	0.28 (0.38)	0.27 (0.3)	0.22 (0.29)
	14-28	0.63 (0.2)	0.46 (0.46)	0.18 (0.32)	0.26 (0.36)	0.3 (0.34)	0.24 (0.32)	0.25 (0.27)	0.22 (0.25)	0.17 (0.19)
	28-57	0.32 (0.66)	0.23 (0.56)	0.32 (0.4)	0.34 (0.39)	0.46 (0.31)	0.39 (0.25)	0.35 (0.19)	0.24 (0.2)	0.15 (0.12)
	57-249	0.96 (0.05)	0.95 (0.04)	0.78 (0.21)	0.85 (0.1)	0.86 (0.07)	0.83 (0.07)	0.78 (0.08)	0.69 (0.1)	0.61 (0.06)
G	0-14	0.96 (0.04)	0.69 (0.32)	0.53 (0.33)	0.84 (0.17)	0.83 (0.09)	0.71 (0.09)	0.68 (0.09)	0.57 (0.08)	0.58 (0.08)
	14-28	0.77 (0.18)	0.85 (0.09)	0.65 (0.26)	0.26 (0.43)	0.59 (0.21)	0.6 (0.18)	0.46 (0.22)	0.31 (0.18)	0.32 (0.14)
	28-57	0.47 (0.53)	0.48 (0.29)	0.42 (0.38)	0.45 (0.3)	0.19 (0.43)	0.38 (0.3)	0.32 (0.28)	0.22 (0.24)	0.16 (0.19)
	57-249	-0.34 (0.96)	0.65 (0.34)	0.19 (0.47)	0.36 (0.39)	0.42 (0.29)	0.33 (0.22)	0.33 (0.23)	0.3 (0.21)	0.28 (0.2)

Values represent the mean cosine similarity (cos θ) calculated from replicate trajectories, with standard deviation shown in parentheses.
Cos θ values close to 1 indicate strong directional consistency between successive trajectory segments, whereas values near 0 or negative indicate directional instability.

3. Supplementary figures

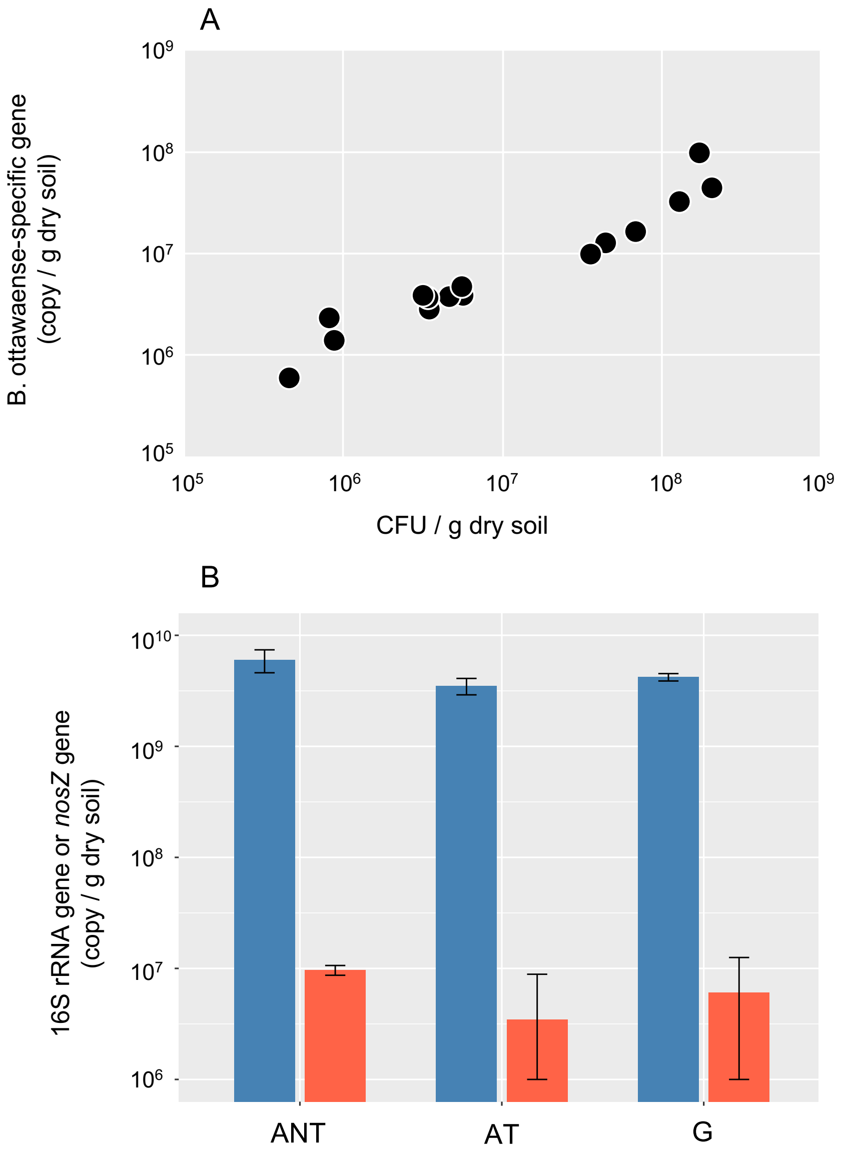

Supplementary Fig. S1. qPCR-based quantification of *B. ottawaense* specific gene, 16S rRNA gene and *nosZ* gene in soil microcosms.
(A) Correlation between viable cell counts and qPCR-based quantification of B. ottawaense in ANT soil. CFU counts *and B. ottawaense*-specific gene copy numbers were measured in ANT soil samples inoculated with SG09_1 across all time points. A significant positive correlation was observed (Pearson's r = 0.857, $p < 0.001$), indicating consistency between culture-based and molecular quantification methods. Each point represents a replicate (n = 3 per time point). (B) Gene abundance of 16S rRNA and *nosZ* in control soils at Day 0. Bars indicate the mean ± standard deviation of triplicate samples (n = 3).

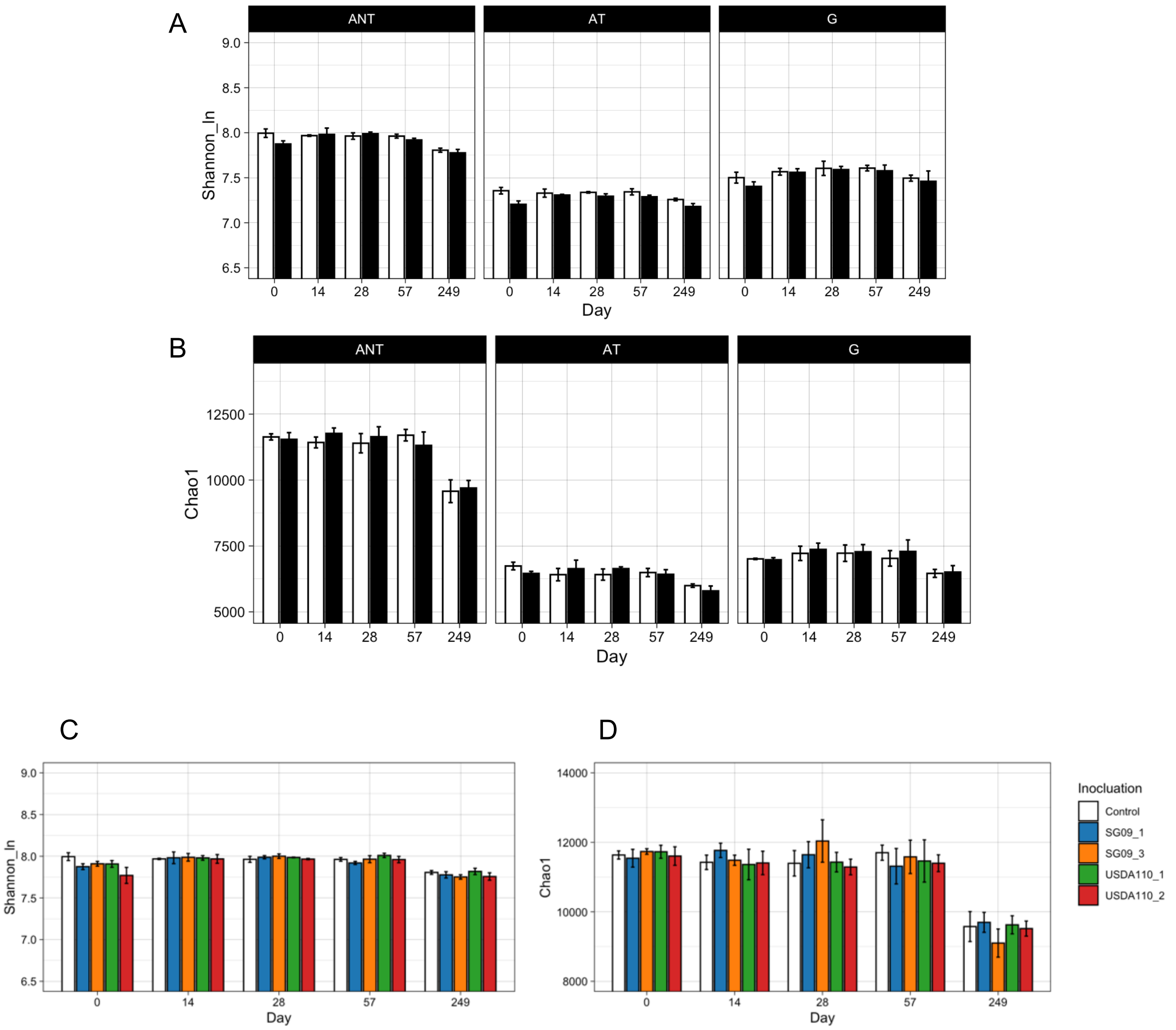

Supplementary Fig. S2. Alpha diversity in control and *Bradyrhizobium*-inoculated soils.
Shannon index (A) and Chao1 richness (B) across time points in ANT, AT, and G soils for uninoculated controls (white bars) and soils inoculated with *B. ottawaense* SG09 (black bars). No significant differences were detected in any soil type or at any time point (Wilcoxon test, BH-adjusted $p > 0.05$).
Shannon index (C) and Chao1 richness (D) in ANT soil comparing four inoculated strains—*B. ottawaense* SG09_1 and SG09_3, or *B. diazoefficiens* USDA110_1 and USDA110_2—with controls. No significant differences were observed at any time point (Dunn's test, BH-adjusted $p > 0.05$). Values represent the means of triplicates, and error bars indicate standard deviations.

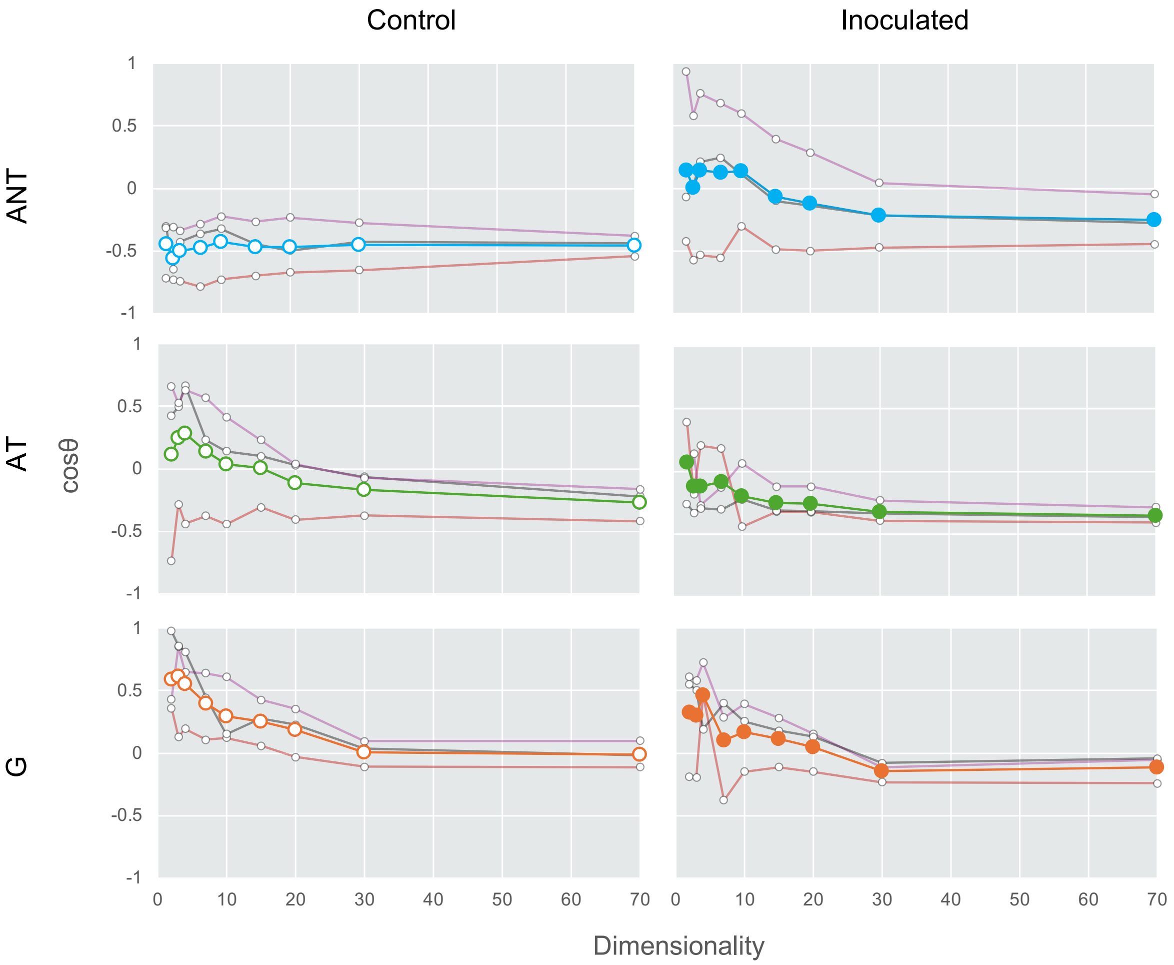

Supplementary Fig. S3. Trajectory smoothness of microbial community trajectories across embedding dimensions. Trajectory smoothness was calculated as the mean cosine similarity (cos θ) between three pairs of adjacent trajectory vectors, defined by successive time intervals (Day 0–14 vs. 14–28, Day 14–28 vs. 28–57, and Day 28–57 vs. 57–249), thereby quantifying local directional consistency of community trajectories across time. Results are shown across increasing embedding dimensions (d = 2–70) for control soils (left panels, open circles) and inoculated soils (right panels, closed circles).

Panels are arranged by soil type (top: ANT; middle: AT; bottom: G). For each condition, individual replicate trajectories (n = 3) are shown as lightly colored points (pale purple, pale red, and gray), and the mean value is indicated by a solid line. Higher cos θ values indicate greater trajectory smoothness, whereas lower or negative values indicate frequent changes in direction.

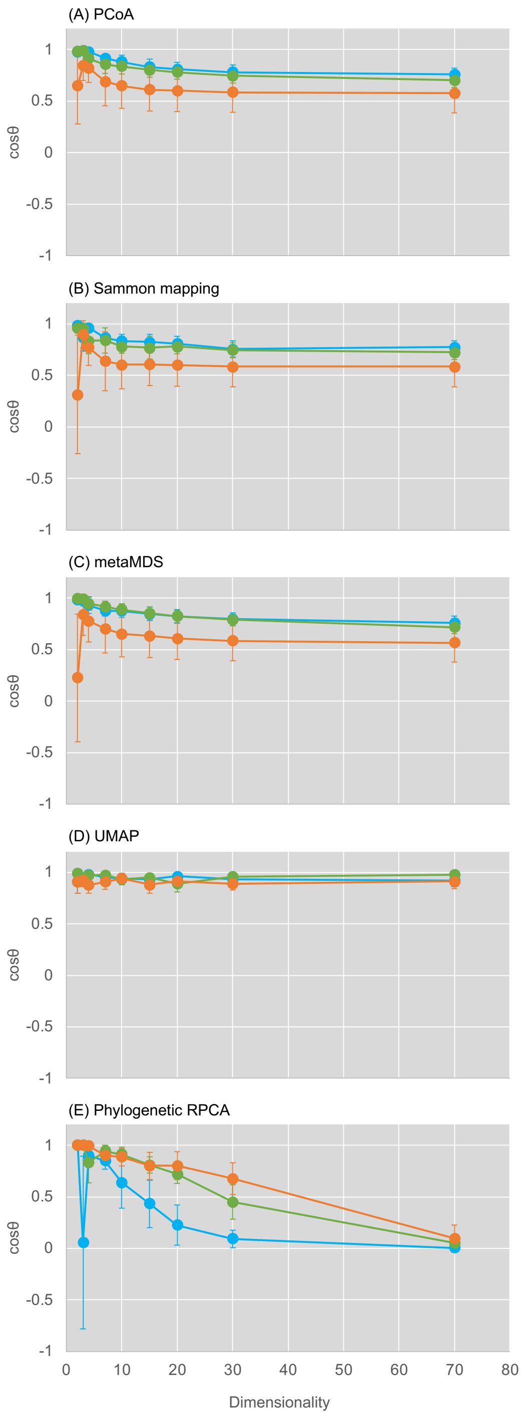

Supplementary Fig. S4. Comparison of mean pairwise cos θ values across multiple ordination methods based on weighted UniFrac distances and phylogenetic RPCA.
Panels A–E show how directional similarity between control and inoculated soils changes with increasing embedding dimension using PCoA (A), Sammon mapping (B), metaMDS (C), UMAP (D), and phylogenetic RPCA (E). For panels A–D, ordination coordinates were derived from weighted UniFrac distance matrices, whereas panel E was obtained from phylogenetic RPCA embeddings. Cos θ values were calculated for each soil type (ANT, AT, and G) by comparing control and inoculated community trajectories from Day 0 to Day 249, and are plotted across increasing embedding dimensions (d = 2, 3, 4, 7, 10, 15, 20, 30, and 70). Error bars represent standard deviations across biological replicates. Blue, green, and red lines correspond to ANT, AT, and G soils, respectively. A cos θ value of 1.0 indicates identical trajectory orientation, 0 indicates orthogonal directions, and –1.0 indicates opposite directions.
Panel B is identical to Fig. 3B in the main text and is included here to facilitate direct comparison with other ordination methods.

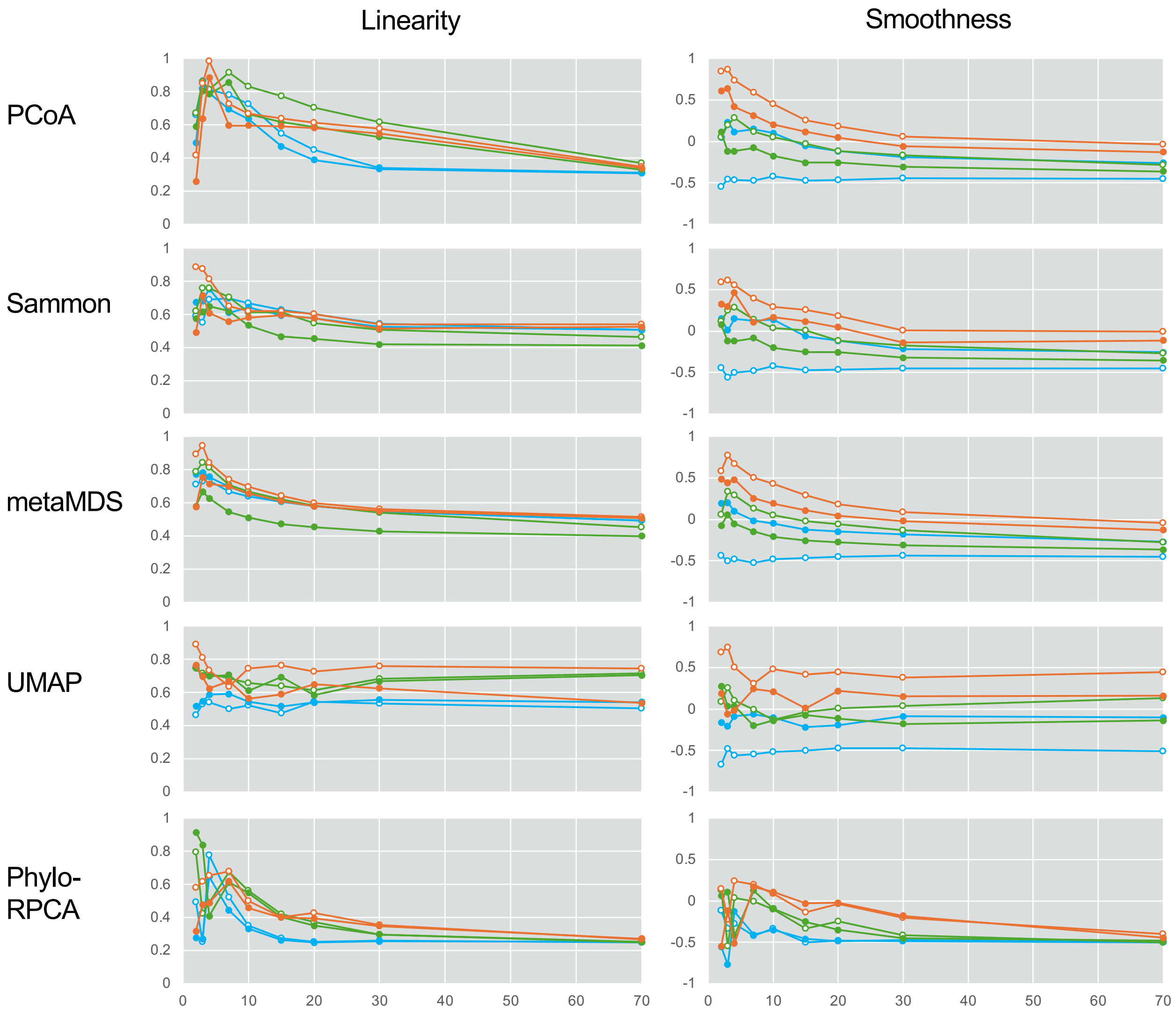

Fig. S5. Comparison of trajectory linearity and smoothness across ordination methods and embedding dimensions.

Trajectory linearity and smoothness were compared across five ordination methods: PCoA, Sammon mapping, metaMDS, and UMAP based on UniFrac distances, and phylogenetic RPCA.

Linearity was calculated as the ratio of the Euclidean distance between the start (Day 0) and end (Day 249) points of a trajectory to the cumulative path length across time points, with higher values indicating straighter global trajectories. Smoothness was calculated as the mean cosine similarity (cos θ) between successive trajectory segments, quantifying local directional consistency across time.

Both metrics were evaluated across increasing embedding dimensions (d = 2–70). Mean values across replicate trajectories (n = 3) are shown for each soil and inoculation treatment. Control soils are shown as open symbols and inoculated soils as closed symbols; soil types are indicated by color (ANT, blue; AT, green; G, orange). Sammon-based linearity and smoothness values correspond to those shown in Fig. 3C and Fig. S4, respectively, and are included here to facilitate comparison among ordination methods.

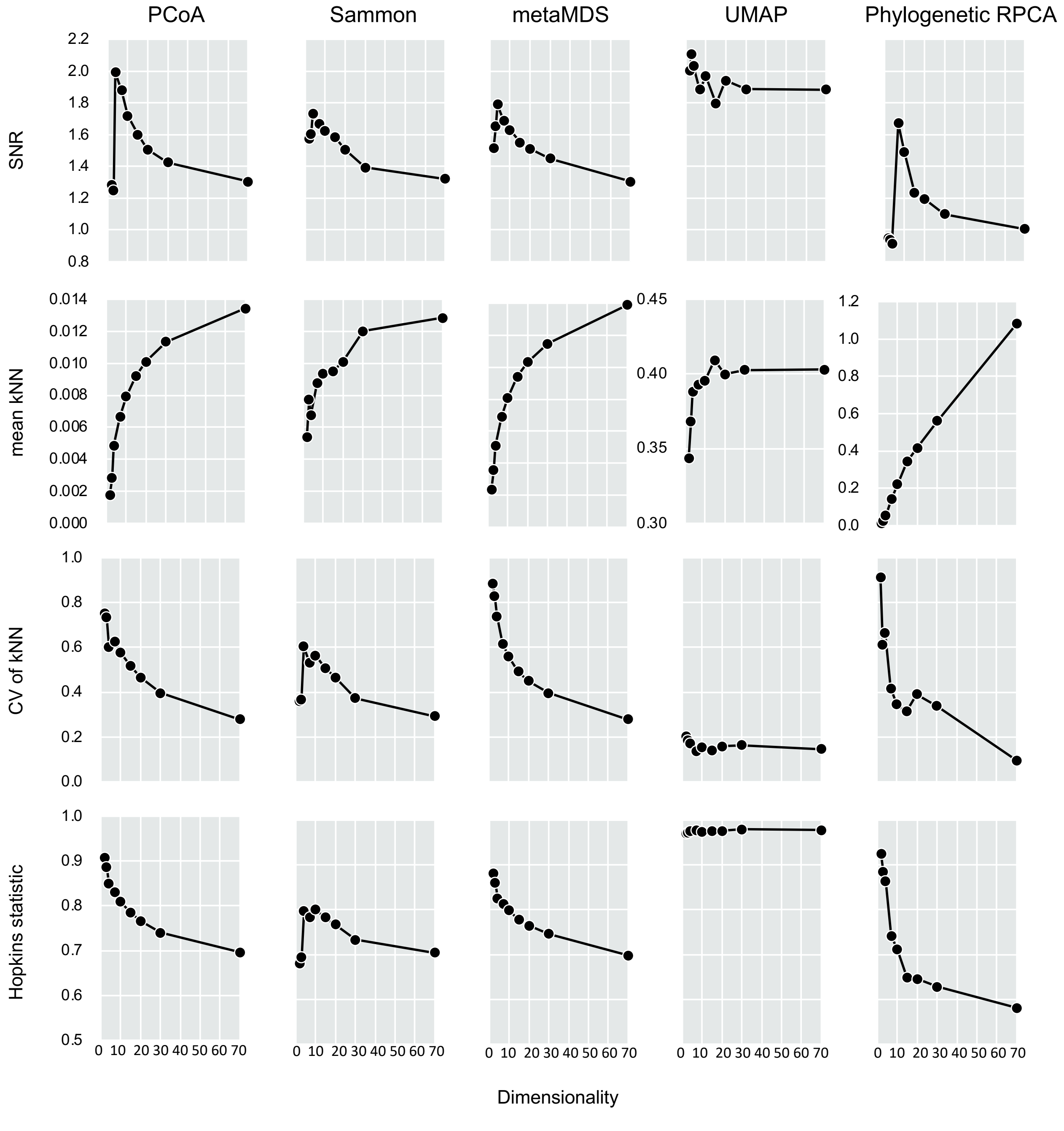

Supplementary Fig. S6. Comparison of clustering-relevant metrics across multiple ordination methods. Clustering-related metrics were compared across PCoA, Sammon mapping, metaMDS, UMAP, and phylogenetic RPCA ordination methods. Metrics include signal-to-noise ratio (SNR), calculated as the ratio of between-treatment (signal) to within-treatment (noise) distances; mean k-nearest neighbor (kNN) distance, representing the degree of local sample sparsity; coefficient of variation (CV) of kNN distances, indicating spatial heterogeneity; and the Hopkins statistic, which quantifies clustering tendency (values closer to 1 indicate stronger clustering, whereas values near 0.5 suggest random distributions). For PCoA, Sammon mapping, metaMDS, and UMAP, metrics were calculated from ordination coordinates derived from weighted UniFrac distance matrices, whereas phylogenetic RPCA metrics were computed from phylogenetic RPCA embeddings based on rCLR-transformed count data. All metrics were evaluated across increasing embedding dimensions (d = 2, 3, 4, 7, 10, 15, 20, 30, and 70).

Sammon results are identical to those presented in Fig. 5 of the main text. Y-axis ranges are generally kept consistent across methods, except where noted (e.g., mean kNN distance in UMAP and Phylogenetic RPCA), to facilitate direct comparison.

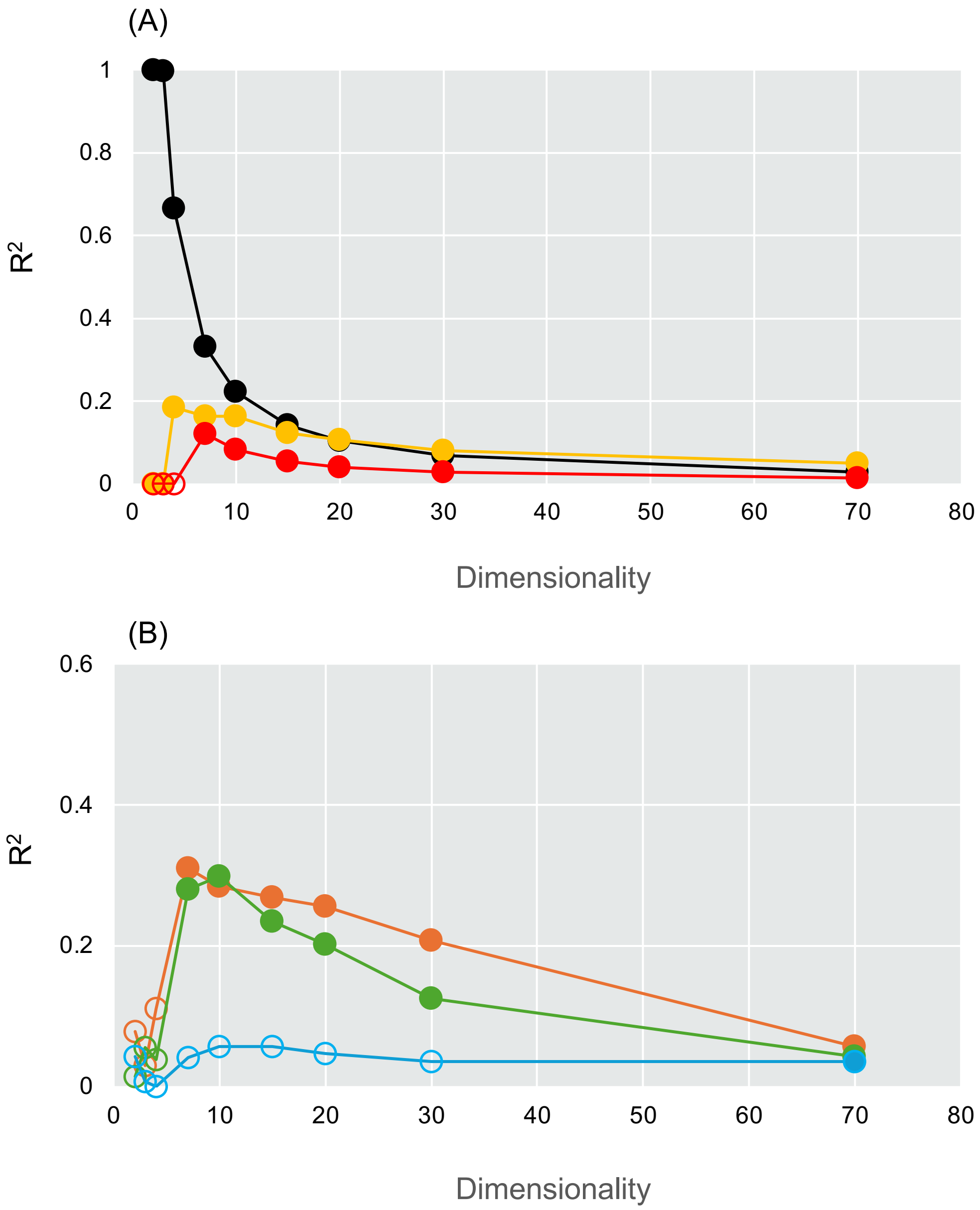

Supplementary Fig. S7. Dimension-dependent PERMANOVA results based on phylogenetic-RPCA-derived distance matrices.
PERMANOVA was performed using distance matrices reconstructed from phylogenetic RPCA embeddings across increasing dimensions (d = 2, 3, 4, 7, 10, 15, 20, 30, and 70).
Panel A shows results from the global model, where the proportion of variance explained (R^2) by soil type (black), time (yellow), and inoculation treatment (red) is plotted against embedding dimensionality.
Panel B shows soil-wise PERMANOVA results, with R^2 values for inoculation treatment plotted separately for ANT (blue), AT (green), and G (orange) soils across dimensions.
Open circles indicate non-significant effects, whereas closed circles indicate statistically significant effects ($p < 0.05$).

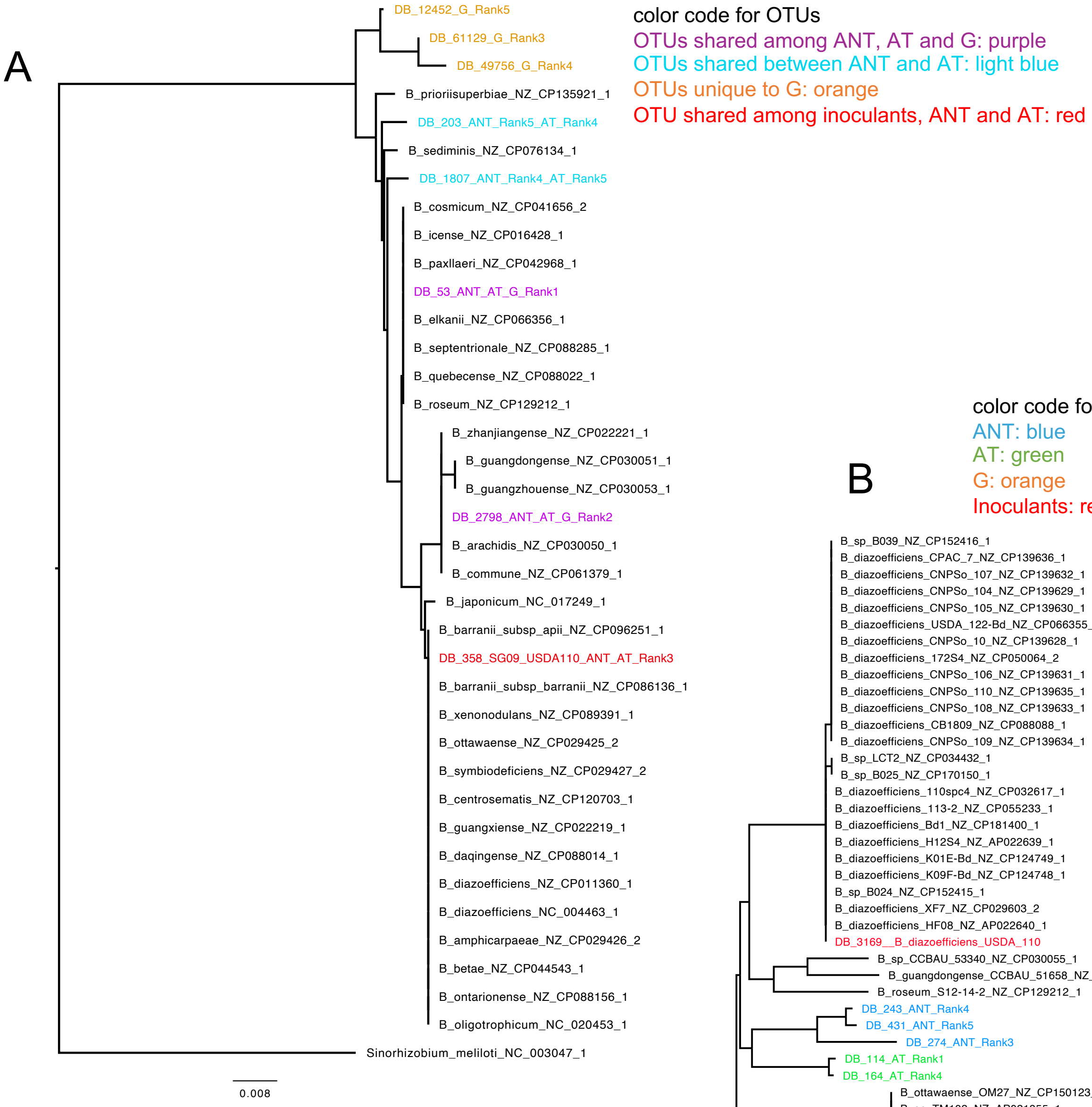

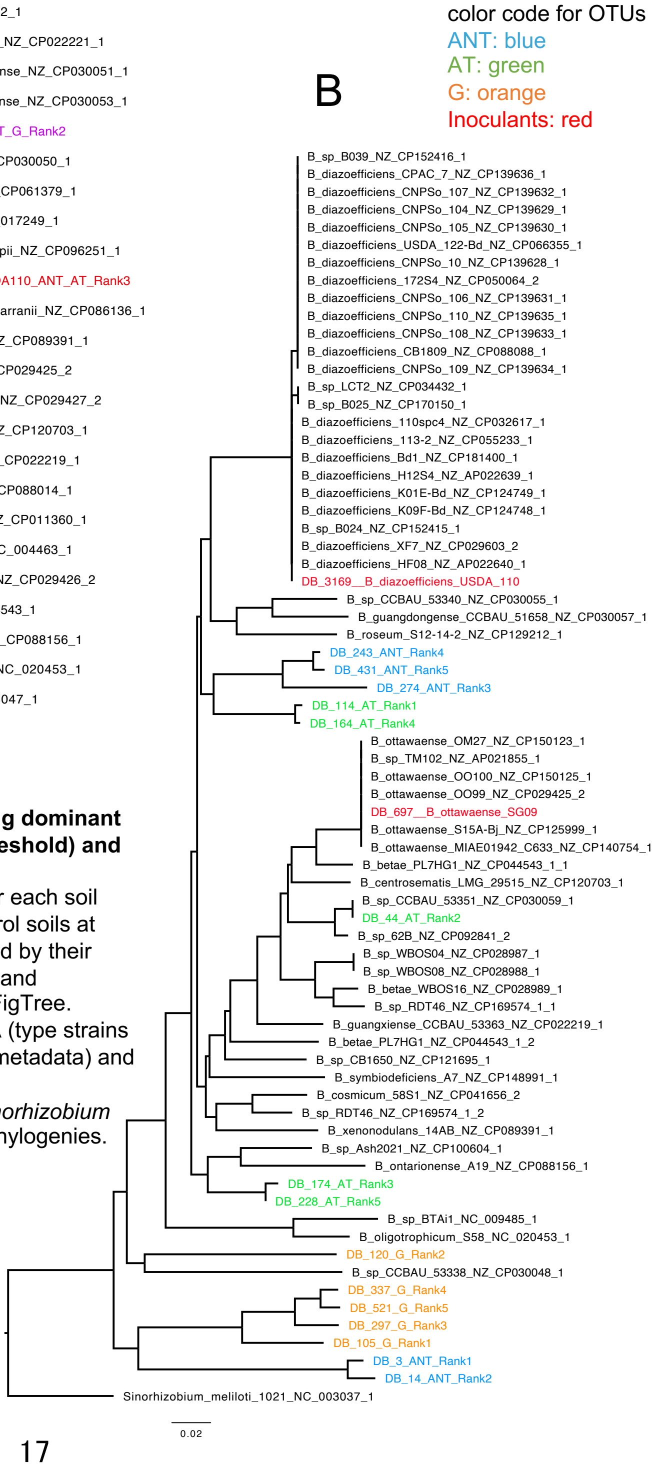

Supplementary Fig. S8. Phylogenetic relationships among dominant *Bradyrhizobium* OTUs (clustered at 1-base mismatch threshold) and reference strains.
(A) 16S rRNA gene and (B) *nosZ* gene-based phylogeny. For each soil type (ANT, AT, and G), the five most abundant OTUs in control soils at Day 0 are shown along with selected reference strains labeled by their accession numbers. Sequences were aligned using MAFFT, and neighbor-joining trees were constructed and visualized with FigTree. Reference sequences were obtained by extracting 16S rRNA (type strains only, as indicated by the type_material qualifier in GenBank metadata) and *nosZ* gene sequences from complete or chromosome-level *Bradyrhizobium* genomes in the NCBI genome database. *Sinorhizobium meliloti* was included as an outgroup in both 16S and *nosZ* phylogenies.

4. Python scripts for computing geometric analysis

4.1 Python script for computing cos θ

```
"""
script1_compute_cos_theta.py

This script calculates the cosine similarity (cosθ) between Control and Inoculated
microbial community trajectories
based on ordination coordinates (e.g., Sammon, PCoA, NMDS, UMAP).
It computes start-to-end vectors (from Day 0 to Day 249) per replicate, and compares
trajectory direction across groups.

The dimensionality (k) is automatically determined based on non-NaN ordination
columns (V1, V2, ..., Vk).

Input:
    A CSV file containing:
        - Ordination coordinates (columns named V1, V2, ..., Vk)
        - Metadata columns:
            - 'soil': categorical variable indicating the soil type
            - 'Day_value': numeric time point (e.g., 0 and 249)
            - 'Replicate': replicate identifier
            - 'treatment': treatment group label ('C' for Control, 'I' for Inoculated)

Output:
    A CSV file with mean cosθ, standard deviation, and the number of pairwise
comparisons for each soil.

Usage:
    python script1_compute_cos_theta.py

Dependencies:
    - pandas
    - numpy
"""

import pandas as pd
import numpy as np
from itertools import product

def calculate_cosine_similarity_control_vs_inoculated_general(csv_path, day_start=0,
day_end=249):
    df = pd.read_csv(csv_path)

    V_cols_all = [c for c in df.columns if c.startswith("V") and c[1:].isdigit()]
    coords = df[V_cols_all].dropna(axis=1, how="all")
    vec_cols = coords.columns.tolist()
    k = len(vec_cols)

    soils = df['soil'].unique()
    results = []

    for s in soils:
        sub = df[df['soil'] == s]
        vectors = {}
        for rep in sub['Replicate'].unique():
            try:
                start = sub[(sub['Replicate'] == rep) & (sub['Day_value'] ==
day_start)][vec_cols].values.squeeze()
                end   = sub[(sub['Replicate'] == rep) & (sub['Day_value'] ==
day_end)][vec_cols].values.squeeze()
                if start.size == 0 or end.size == 0:
                    continue
                vectors[rep] = end - start
            except Exception:
                continue

        rep_treatments = sub.groupby("Replicate")["treatment"].first().to_dict()
        control_reps    = [r for r in vectors if rep_treatments.get(r) == "C"]
        inoculated_reps = [r for r in vectors if rep_treatments.get(r) == "I"]

        cos_vals = []
        for r1 in control_reps:
            for r2 in inoculated_reps:
                a, b = vectors[r1], vectors[r2]
                if np.linalg.norm(a) == 0 or np.linalg.norm(b) == 0:
```

```python
                    continue
                cos = np.dot(a, b) / (np.linalg.norm(a) * np.linalg.norm(b))
                cos_vals.append(cos)

        mean_cos = np.mean(cos_vals) if len(cos_vals) else np.nan
        sd_cos   = np.std(cos_vals, ddof=1) if len(cos_vals) > 1 else np.nan

        results.append({
            'soil': s,
            'k': k,
            'mean_cos': mean_cos,
            'sd_cos': sd_cos,
            'n_pairs': len(cos_vals)
        })

    return pd.DataFrame(results)


if __name__ == "__main__":
    input_file = "<input_file.csv>"   # Replace with your CSV file path
    result_df = calculate_cosine_similarity_control_vs_inoculated_general(input_file)
    output_file = "pairwise_cosine.csv"
    result_df.to_csv(output_file, index=False)
    print(f"Output saved to: {output_file}")
```

4.2 Python script for computing SNR

```python
"""
script2_compute_snr.py

This script computes Signal-to-Noise Ratio (SNR) from multiple ordination coordinate
files (e.g., PCoA, Sammon, NMDS, UMAP) by calculating within- and between-group
Euclidean distances.

Usage:
    script2_compute_snr.py --input_file input.csv --output_file result.csv

Dependencies:
    - pandas
    - numpy
"""

import argparse
import logging
import os
from itertools import combinations

import numpy as np
import pandas as pd

logging.basicConfig(level=logging.INFO, format="%(levelname)s: %(message)s")


def compute_snr(df, coord_cols):
    """
    df        : DataFrame including 'soil', 'treatment', 'Day' and ordination columns
(V1..Vk)
    coord_cols : list of ordination column names

    Returns:
        (snr, mean_within, mean_between) or None if not computable
    """

    # ----- Cleaning step (Strategy A) -----
    coords_raw = df[coord_cols].replace([np.inf, -np.inf], np.nan)

    n_cols_before = coords_raw.shape[1]
    n_rows_before = coords_raw.shape[0]

    # Drop columns with any NaN (original behavior)
    coords = coords_raw.dropna(axis=1)
    dropped_cols = n_cols_before - coords.shape[1]

    # Drop remaining NaN rows (minimum necessary)
    valid_rows = coords.notna().all(axis=1)
    coords = coords.loc[valid_rows]
    df = df.loc[valid_rows]
    dropped_rows = n_rows_before - coords.shape[0]

    # NaN/inf check
    n_nan = np.isnan(coords.values).sum()
    n_inf = np.isinf(coords.values).sum()
```

```
    logging.debug(
        f"After cleaning: coords.shape={coords.shape}, "
        f"dropped_cols={dropped_cols}, dropped_rows={dropped_rows}, "
        f"NaN cells={n_nan}, inf cells={n_inf}"
    )

    # Dimension check
    if coords.shape[1] < 2 or coords.shape[0] < 2:
        return None

    # ----- Distance matrix (NumPy implementation to avoid sklearn warnings) -----
    X = coords.to_numpy(dtype=float)         # (n, k)
    diff = X[:, None, :] - X[None, :, :]      # (n, n, k)
    dist_matrix = np.sqrt(np.einsum('ijk,ijk->ij', diff, diff))  # (n, n)

    # ----- Original SNR logic -----
    replicate_groups = df.groupby(["soil", "treatment", "Day"]).groups

    # within-group distances
    within = [
        dist_matrix[i, j]
        for group in replicate_groups.values()
        for i, j in combinations(group, 2)
    ]

    # between-group distances
    between = []
    for _, row in df[["soil", "Day"]].drop_duplicates().iterrows():
        mask = (df["soil"] == row["soil"]) & (df["Day"] == row["Day"])
        subset = df[mask]
        treatments = subset["treatment"].unique()
        if len(treatments) < 2:
            continue
        for t1, t2 in combinations(treatments, 2):
            idx1 = subset[subset["treatment"] == t1].index
            idx2 = subset[subset["treatment"] == t2].index
            between.extend(dist_matrix[i, j] for i in idx1 for j in idx2)

    mean_within = np.nanmean(within) if within else np.nan
    mean_between = np.nanmean(between) if between else np.nan
    snr = (
        mean_between / mean_within
        if mean_within and not np.isclose(mean_within, 0.0)
        else np.nan
    )

    return snr, mean_within, mean_between


def main(input_file, output_file):
    try:
        df = pd.read_csv(input_file)
        required = {"soil", "treatment", "Day"}
        missing = required - set(df.columns)
        if missing:
            raise ValueError(f"Missing required columns: {missing}")

        coord_cols = [c for c in df.columns if c.startswith("V")]
        if not coord_cols:
            raise ValueError("No ordination columns found (expected columns starting
with 'V').")

        snr_result = compute_snr(df, coord_cols)
        if snr_result:
            snr, within, between = snr_result
            result = pd.DataFrame(
                [
                    {
                        "filename": os.path.basename(input_file),
                        "SNR": snr,
                        "mean_within": within,
                        "mean_between": between,
                    }
                ]
            )
            result.to_csv(output_file, index=False)
            logging.info(f"Saved result to {output_file}")
        else:
            logging.warning("Insufficient dimensions or rows after cleaning. No result
written.")

    except Exception as e:
        logging.error(f"Failed to process {input_file}: {e}")


if __name__ == "__main__":
```

```
    parser = argparse.ArgumentParser()
    parser.add_argument("--input_file", required=True, help="Path to the input CSV
file")
    parser.add_argument("--output_file", required=True, help="Path to output CSV
file")
    args = parser.parse_args()
    main(args.input_file, args.output_file)
```

4.3 Python script for computing kNN

```
"""
script3_compute_kNN.py

This script computes the mean distance to k nearest neighbors (kNN) and the
coefficient of variation (CV)
from multiple ordination coordinate files (e.g., PCoA, Sammon, NMDS, UMAP). For each
file in the input folder,
the script calculates distances to the 5 nearest neighbors (excluding self) for each
sample, then reports
the overall mean kNN distance and its coefficient of variation across samples.

Input:
    - A folder containing ordination CSV files.
      Each file must include ordination coordinate columns named V1, V2, ..., Vk.
      (Other metadata columns will be ignored.)

Output:
    - A summary CSV file listing, for each input file:
        - filename
        - number of dimensions (k)
        - mean kNN distance
        - coefficient of variation (CV) of kNN distances

Usage:
    python script3_compute_kNN.py

Dependencies:
    - pandas
    - numpy
    - scikit-learn
"""

import pandas as pd
import numpy as np
from sklearn.neighbors import NearestNeighbors
import os
import glob

# Input folder and output file path
input_folder = "/path/to/your/input_data"  # Modify as needed
output_file = "./kNN_summary.csv"

# List to store results
results = []

# Process all CSV files in the folder
for file_path in glob.glob(os.path.join(input_folder, "*.csv")):
    try:
        df = pd.read_csv(file_path)

        # Extract coordinate columns (exclude columns with NaN)
        coord_cols = [col for col in df.columns if col.startswith('V')]
        coords = df[coord_cols].dropna(axis=1).values

        # Check number of dimensions (skip if insufficient)
        if coords.shape[1] < 2:
            continue

        # Calculate kNN distances
        nn = NearestNeighbors(n_neighbors=6).fit(coords)
        distances, _ = nn.kneighbors(coords)
        d_kNN = distances[:, 1:6].mean(axis=1)  # Exclude distance to self
        mean_kNN = d_kNN.mean()
        cv_kNN = d_kNN.std() / mean_kNN if mean_kNN != 0 else np.nan

        # Save results
        results.append({
            "file": os.path.basename(file_path),
            "dimensions": coords.shape[1],
            "mean_kNN": mean_kNN,
            "cv_kNN": cv_kNN
        })
```

```
    except Exception as e:
        print(f"Error processing {file_path}: {e}")

# Output results as DataFrame to CSV
summary_df = pd.DataFrame(results)
summary_df.to_csv(output_file, index=False)
```

224

225 ## 4.4 Python script for computing Hopkins statistic

```
"""
script4_compute_hopkins.py

This script computes the Hopkins statistic to assess the clustering tendency of
samples
based on ordination coordinate files (e.g., PCoA, Sammon, NMDS, UMAP). For each input
CSV file,
ordination coordinates (V1..Vk) are extracted, and the Hopkins statistic is
calculated by
random sampling (default 1000 replicates). The mean Hopkins value across replicates
is reported.

Input:
   - A directory containing ordination CSV files.
     Each file must include ordination coordinate columns named V1, V2, ..., Vk.
     (Other metadata columns will be ignored.)

Output:
   - A summary CSV file with, for each input file:
       - filename
       - number of dimensions (k)
       - mean Hopkins statistic across replicates

Usage:
   python script4_compute_hopkins.py <input_directory>

Dependencies:
   - pandas
   - numpy
   - scikit-learn
"""

import os
import pandas as pd
import numpy as np
from sklearn.neighbors import NearestNeighbors
import argparse

# ---- Hopkins calculation function ----
def hopkins_statistic(X, m=50):
    n, d = X.shape
    if n < m:
        m = n
    idx = np.random.choice(np.arange(n), m, replace=False)
    X_sample = X[idx]
    mins = np.min(X, axis=0)
    maxs = np.max(X, axis=0)
    U = np.random.uniform(mins, maxs, size=(m, d))
    nbrs = NearestNeighbors(n_neighbors=2).fit(X)
    distances_w, _ = nbrs.kneighbors(X_sample, n_neighbors=2)
    w = distances_w[:, 1]
    distances_u, _ = nbrs.kneighbors(U, n_neighbors=1)
    u = distances_u[:, 0]
    return u.sum() / (u.sum() + w.sum())

# ---- Argument Parsing ----
parser = argparse.ArgumentParser()
parser.add_argument("input_dir", help="Input directory containing CSV files")
args = parser.parse_args()

# ---- Processing Each CSV ----
results = []
for filename in os.listdir(args.input_dir):
    if filename.endswith(".csv"):
        filepath = os.path.join(args.input_dir, filename)
        df = pd.read_csv(filepath)
        coord_cols = [col for col in df.columns if col.startswith("V")]
        coords = df[coord_cols].dropna(axis=1).values
        if coords.shape[0] < 2 or coords.shape[1] < 1:
            continue
        hopkins_vals = [hopkins_statistic(coords) for _ in range(1000)]
        hopkins_mean = np.mean(hopkins_vals)
        hopkins_min = np.min(hopkins_vals)
        hopkins_max = np.max(hopkins_vals)
```

```
        hopkins_std = np.std(hopkins_vals)
        results.append({
            "file": filename,
            "dimensions": coords.shape[1],
            "hopkins_mean": hopkins_mean,
            "hopkins_min": hopkins_min,
            "hopkins_max": hopkins_max,
            "hopkins_std": hopkins_std
        })

# ---- Save Results ----
output_csv_path = os.path.join(args.input_dir, "hopkins_results.csv")
results_df = pd.DataFrame(results)
results_df.to_csv(output_csv_path, index=False)
```
